# The impact of slippage on the data quality of head-worn eye trackers

**DOI:** 10.3758/s13428-019-01307-0

**Published:** 2020-01-02

**Authors:** Diederick C. Niehorster, Thiago Santini, Roy S. Hessels, Ignace T. C. Hooge, Enkelejda Kasneci, Marcus Nyström

**Affiliations:** 1 Lund University Humanities Laboratory and Department of Psychology, Lund University, Lund, Sweden; 2 Human Computer Interaction, University of Tübingen, Tübingen, Germany; 3 Experimental Psychology, Helmholtz Institute and Developmental Psychology, Utrecht University, Utrecht, The Netherlands; 4 Experimental Psychology, Helmholtz Institute, Utrecht University, Utrecht, The Netherlands; 5 Lund University Humanities Laboratory, Lund University, Lund, Sweden

**Keywords:** Head-mounted eye tracking, Wearable eye tracking, Mobile eye tracking, Eye movements, Natural behavior, Data quality

## Abstract

Mobile head-worn eye trackers allow researchers to record eye-movement data as participants freely move around and interact with their surroundings. However, participant behavior may cause the eye tracker to slip on the participant’s head, potentially strongly affecting data quality. To investigate how this eye-tracker slippage affects data quality, we designed experiments in which participants mimic behaviors that can cause a mobile eye tracker to move. Specifically, we investigated data quality when participants speak, make facial expressions, and move the eye tracker. Four head-worn eye-tracking setups were used: (i) Tobii Pro Glasses 2 in 50 Hz mode, (ii) SMI Eye Tracking Glasses 2.0 60 Hz, (iii) Pupil-Labs’ Pupil in 3D mode, and (iv) Pupil-Labs’ Pupil with the Grip gaze estimation algorithm as implemented in the EyeRecToo software. Our results show that whereas gaze estimates of the Tobii and Grip remained stable when the eye tracker moved, the other systems exhibited significant errors (0.8–3.1^∘^ increase in gaze deviation over baseline) even for the small amounts of glasses movement that occurred during the speech and facial expressions tasks. We conclude that some of the tested eye-tracking setups may not be suitable for investigating gaze behavior when high accuracy is required, such as during face-to-face interaction scenarios. We recommend that users of mobile head-worn eye trackers perform similar tests with their setups to become aware of its characteristics. This will enable researchers to design experiments that are robust to the limitations of their particular eye-tracking setup.

**Electronic supplementary material:**

The online version of this article (10.3758/s13428-019-01307-0) contains supplementary material, which is available to authorized users.

## Introduction

In the last decades, mobile (head-worn) eye trackers have become a popular research tool after the pioneering work of researchers such as Michael Land (e.g., Land 1992; Land & Lee 1994; Land, Mennie, & Rusted, 1999) and Dana Ballard, Mary Hayhoe, and Jeff Pelz (e.g., Ballard, Hayhoe, & Pelz, 1995; Pelz & Canosa 2001). Compared to tower-mounted and remote eye trackers, which typically constrain participants to a chair and possibly a chin rest, head-worn eye trackers allow recording eye movements from participants that freely move around. This enables many studies that are not feasible with screen-based eye trackers, such as decision-making research in supermarkets (Gidlöf, Wallin, Dewhurst, & Holmqvist, 2013; Gidlöf, Anikin, Lingonblad, & Wallin, 2017), viewing behavior of medical professionals (Dik, Hooge, van Oijen, & Siersema, 2016), shared manipulation in human–robot interaction (Aronson et al., 2018), foot placement in difficult terrain (Matthis, Yates, & Hayhoe, 2018), visual behavior of teachers in a classroom (McIntyre, Jarodzka, & Klassen, 2017; McIntyre & Foulsham, 2018), as well as dyadic interaction between adults (Ho, Foulsham, & Kingstone, 2015; Rogers, Speelman, Guidetti, & Longmuir, 2018; Macdonald & Tatler 2018; although some interaction studies have been performed with remote eye trackers, see Hessels, Cornelissen, Hooge, & Kemner, 2017; Hessels, Holleman, Kingstone, Hooge, & Kemner, 2019) or children and their parents (Yu & Smith 2017; Suarez-Rivera, Smith, & Yu, 2019).

Little is known however about the data quality of head-worn eye-tracking setups. This is in stark contrast to remote eye tracking, where several studies have called attention to the characterization of eye-tracking data quality (Blignaut & Wium^2014^; Wass, Forssman, & Leppänen, 2014; Nyström, Andersson, Holmqvist, & van de Weijer, 2013; Hessels, Andersson, Hooge, Nyström, & Kemner, 2015), with some studies specifically examining data quality using a series of tests mimicking participant behavior during typical recording sessions (Hessels, Cornelissen, Kemner, & Hooge, 2015; Niehorster, Cornelissen, Holmqvist, Hooge, & Hessels, 2018). Although it has been established that eye camera positioning and illumination conditions can greatly influence tracking quality in head-worn eye tracking (Świrski, Bülling, & Dodgson, 2012; Tonsen, Zhang, Sugano, & Bülling, 2016; Fuhl, Tonsen, Bülling, & Kasneci, 2016), to the best of the authors’ knowledge, only a single study has actually empirically compared the accuracy and precision of multiple head-worn eye-tracking setups—yet the study (MacInnes, Iqbal, Pearson, & Johnson, 2018) was limited in scope to the ideal case of careful calibration and evaluation immediately thereafter. It is therefore not representative of how these eye-tracking setups are often used with unconstrained participants in uncontrolled environments. Furthermore, two studies reporting on the accuracy and precision of a single eye-tracking setup are available (Schwaller, 2014; Schüssel et al., 2016) as well as a study reporting on the accuracy achieved with unconstrained participants in mobile eye-tracking recordings performed using another single eye-tracking setup (Santini et al., 2018). As such, while head-worn eye trackers ostensibly enable recording the looking behavior of people during many daily activities, it is not known whether common head-worn eye-tracking setups actually provide gaze position data of sufficient quality in these situations to be viable tools for scientific research. An example of insufficient data quality would be if the eye-tracking setup records gaze positions with a systematic offset that is too large to reliably distinguish which of two nearby objects of interest an observer looks at (Orquin & Holmqvist 2018; Hessels, Kemner, van den Boomen, & Hooge 2016), such as for instance different facial features.

Eye-tracker manufacturers often limit themselves to reporting data-quality measurements for well-controlled and optimal scenarios, and many researchers simply reiterate the manufacturer’s statements as applicable to their studies instead of assessing data quality of the eye-tracking setup as used in their study (see e.g., Wang et al., 2019; Freeth & Bugembe 2018; Hoppe, Loetscher, Morey, & Bülling, 2018). Also a recent overview of head-worn eye-tracking setups (Cognolato, Atzori, & Müller, 2018) compared eye-tracker performance based on manufacturer-provided data-quality values instead of measuring these values themselves. These practices might lead researchers to overestimate performance when choosing an eye-tracking setup. Critically, these practices also make it difficult to evaluate whether data quality in a study was sufficient to support the analysis performed and the resulting conclusions. Measuring, understanding, and reporting the characteristics of an eye-tracking setup is of critical importance both when designing a study and when interpreting the recorded data.

**Fig. 1:**
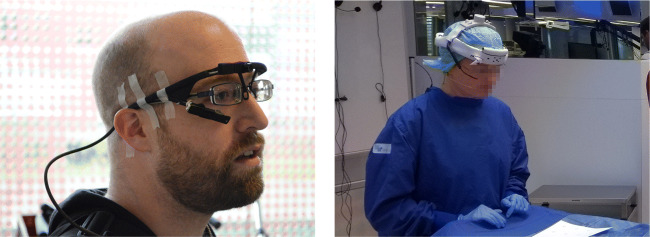
Head-worn eye trackers. Two examples of extreme measures taken by researchers to prevent slippage of a head-worn eye tracker: tape (*left panel*, courtesy of Jeroen Benjamins) and a helmet mount (*right panel*, courtesy of Shahram Eivazi)

Head-worn eye trackers can move with respect to the participant’s head during a recording (Kolakowski & Pelz, 2006). This ‘slippage’ has been found to be the main reason that accuracy during a long recording with unconstrained participants markedly deteriorated (Santini et al., 2018), has led manufacturers to implement drift correction procedures (e.g., SR-Research’s EyeLink 2), and slippage-induced data-quality problems have been one of the reasons why some researchers decided to discard their eye-tracking data altogether (Birmingham, Johnston, & Iarocci, 2017). Personal observations indicate that even when the manufacturer’s design includes tight headbands and other measures to prevent slippage, head-worn eye trackers often do not stay in place during a recording. Dedicated experimenters are required to take extreme measures to alleviate the issue (Fig. 1). Eye-tracker slippage occurs for instance when participants push the head-worn eye tracker back up on their nose, or when they take it off to rub their eyes or adjust their hair. Furthermore, even when the participant does not touch the eye tracker, movement of the facial muscles such as when speaking or making facial expressions may cause the eye tracker to move. Recently, multiple vendors (Tobii and Pupil-labs) have advertised their latest head-worn eye-tracking setups as being robust to device slippage. Due to the lack of tests of this claim, it is however unknown whether these eye-tracking setups indeed provide high data quality when they slip.

In this paper, we test how different head-worn eye-tracking setups perform during a series of conditions designed to mimic slippage-inducing situations that may occur during natural participant behavior. Note that we examine the performance of complete eye-tracking setups, i.e., the combination of eye-tracker hardware and software. We use the term *eye-tracking setup* to refer to these complete setups, and we use the terms *eye tracker* or *headset* when referring to only the hardware. We compared the performance of four head-worn eye-tracking setups. Three setups were chosen because they constitute popular out-of-the-box eye-tracking setups, namely: (1) the Tobii Pro Glasses 2 at 50 Hz, (2) the SMI Eye Tracking Glasses (ETG) 2.0 at 60 Hz, and (3) the Pupil-labs monocular Pupil headset (Cam 1 camera models) together with the Pupil Capture software in default 3D mode.

Open hardware platforms that provide unrestricted access to the cameras in the headset, such as those offered by Pupil-labs, Dikablis and Positive Science, can however readily be used with other recording software and gaze estimation methods to form new eye-tracking setups. These new eye-tracking setups can be designed for a specific situation faced by the experimenter, such as slippage robustness in the case of this paper. As an example of such bespoke eye-tracking setups, we include a fourth setup that also uses the Pupil headset, but replaces the Pupil Capture software with the open-source EyeRecToo software (Santini, Fuhl, Geisler, & Kasneci, 2017). EyeRecToo was chosen for this setup because (1) it is readily available as a pre-compiled executable, (2) previous studies suggest that its tracking algorithms are state-of-the-art (Santini, Fuhl, & Kasneci, 2018b), and (3) it provides the *Grip* (Santini, Niehorster, & Kasneci, 2019) gaze estimation method, which is claimed to be slippage-robust. We will henceforth refer to this setup as *Grip*.

Performance of these head-worn eye-tracking setups was examined as follows. Immediately after eye-tracking setup calibration and a nine-point validation recording, participants were instructed to (1) *speak*, (2) *make* facial expressions, (3) *move* the head-worn eye tracker repeatedly with their hands by small amounts in different directions, and (4) *readjust* the eye tracker back to the calibration position. These conditions are designed to evoke movements of the eye tracker with respect to the participant’s eyes, which we will call *slippage* in this article. These conditions were used to assess whether the gaze position signal provided by an eye-tracking setup is affected by slippage. A setup for which the gaze position signal is unaffected by eye-tracker slippage will be referred to as a *slippage-robust setup* in this article.

The sequence of eye-tracker slippage conditions was preceded and succeeded by nine-point validation conditions to assess post-calibration data quality as well as data quality after participants attempted to put the eye tracker back in the same place it was during calibration, to simulate the eye tracker being taken off and put back in place.

To determine the data quality of the head-worn eye-tracking setups, we will use three concepts commonly used in the literature (Holmqvist, Nyström, & Mulvey, 2012; McConkie 1981): accuracy, precision, and data loss. Putting the definitions of these terms in the International Vocabulary of Metrology (BIPM et al., 2012) in the context of eye-tracking data, accuracy is the closeness of the gaze position reported by the eye-tracking setup to the actual gaze position of the participant, and precision is the closeness of a set of reported gaze positions. Data loss is the relation between the number of measurements achieved by an eye-tracking setup in a given time interval to the number of measurements that should be expected based on the specifications of the eye-tracking setup.

This study provides the reader with (a) a logic for designing tests of head-worn eye-tracking setups that are relevant to their planned or current usage of these devices, as well as (b) a practical insight into what data quality can be expected from a selection of prominent head-worn eye-tracking setups during unconstrained participant behavior. This article is meant to raise awareness about that eye-tracking data recorded with head-worn eye trackers may not be of sufficient quality when recording from freely moving participants and that researchers therefore must *themselves* test their setup to determine the data quality they may expect from their recordings. We therefore furthermore discuss the effect of ignoring the characteristics and limitations of head-worn eye-tracking setups on the validity of studies conducted with them. In our experience with many eye-tracking projects and when teaching eye tracking to users from a wide variety of research fields, this is an important insight for users of eye trackers. The current study should be taken as a blueprint for how to scientifically assess whether an eye-tracking setup is suitable for the researcher’s study design, and is expected to spur researchers to perform such tests themselves.

## Method

### Participants

Nine volunteers (eight naïve to the specific goals of the study and author MN; seven males, two females) between the ages of 28 and 42 years participated in the experiment at Lund University. All participants had normal or corrected-to-normal vision and provided informed consent. None wore glasses or had eye lids or eye lashes that occluded the pupil.

### Stimuli

**Fig. 2:**
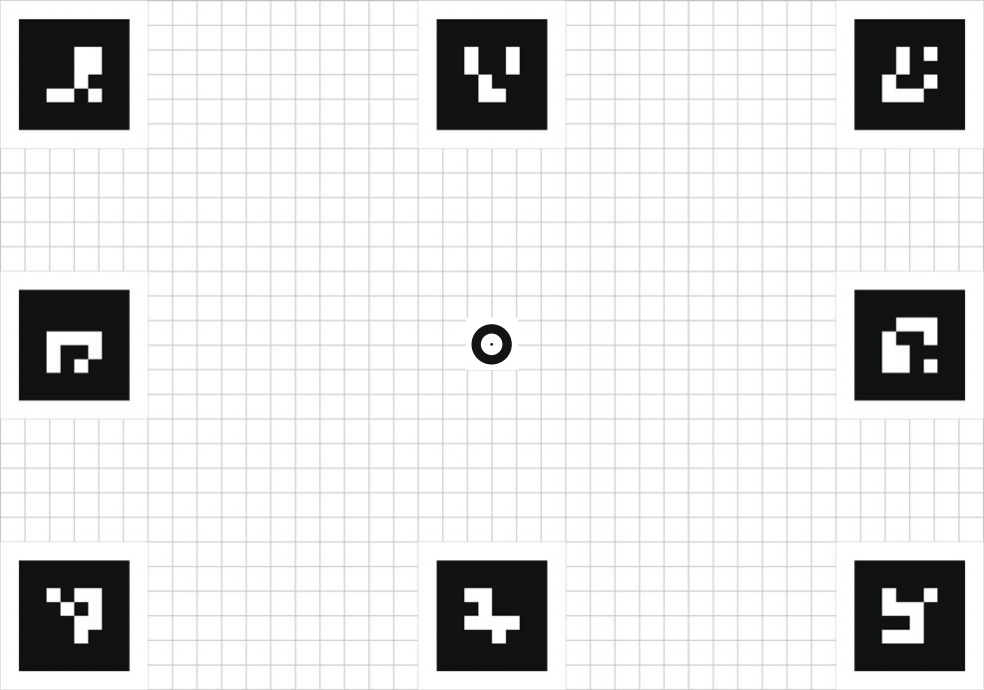
The stimulus grid. Stimulus grid for the Tobii, containing a Tobii calibration target at the center and eight ArUco markers at the corners and along the edges. For the other eye-tracking setups, different center markers were used. The stimulus grid used for our experiments spanned (horizontally and vertically) 105 × 73 cm (equivalent to 40 × 28^∘^ when viewed from a distance of 1.5 m)

In a room lit by fluorescent tubes, participants stood 1.5 m away from a large paper sheet on which a stimulus grid was printed (Fig. 2). The grid spanned (horizontally and vertically) 105 × 73 cm (equivalent to 40 × 28^∘^ when viewed from a distance of 1.5 m). Eight ArUco markers (Garrido-Jurado, Munoz-Salinas, Madrid-Cuevas, & Medina-Carnicer, 2016) were placed at the corners and the midpoints of the sides of the stimulus grid. These markers along with the centrally located calibration marker provided gaze targets for the participants, and a means for automatic data analysis by enabling mapping of gaze position in the scene video to the plane extending from the stimulus grid. At the center of the stimulus grid, different calibration targets were put in place depending on the eye-tracking setup. Full-size print-ready PDF versions of the stimulus grid with the Tobii, Pupil-labs single-marker or EyeRecToo calibration targets are available online in the Supplementary Material (SMI does not utilize a predefined calibration target).

### Apparatus

In this section, we describe the hardware, software, and calibration employed for each eye-tracking setup, including versions and configurations. Recordings were made with the following four head-worn eye-tracking setups:

**Tobii**: This setup used the Tobii Pro Glasses 2, consisting of a head-worn eye tracker connected to a recording unit (firmware version 1.25.3-citronkola). This is a binocular eye-tracking setup using two cameras and six glints per eye for gaze tracking and was chosen because it is commonly used (e.g., Rogers et al., 2018; Raptis, Fidas, & Avouris, 2018) and because Tobii explicitly claims that their product is slippage-robust.^1^
  The recording unit was connected to a laptop running the Tobii Glasses controller software (version 1.95) using an Ethernet cable. The system was set to 50 Hz mode, and calibrated using its one-point calibration procedure using the marker provided with the eye-tracking setup. The calibration consisted of (a) fixing this marker to the center of the stimulus grid, (b) instructing the participant to fixate the marker’s center, and (c) entering calibration mode in the Tobii software, after which the process completed automatically. The front-facing scene camera recorded a video stream at 25 Hz with a 1920 × 1080 px resolution, and the four eye cameras recorded a stream at 50 Hz containing four eye images (two views for each eye) with a combined resolution of 240 × 960 px.
**SMI**: This setup used the SensoMotoric Instruments (SMI) Eye Tracking Glasses 2.0 60 Hz and an SMI-provided laptop. This is a binocular eye-tracking setup using one camera and six glints per eye for gaze tracking. Although the SMI system is no longer available for sale, we chose to include it because a significant number of these head-worn eye-tracking setups have been sold and they are still commonly used in mobile eye-tracking studies (e.g., Ahlstrom, Kircher, Thorslund, & Adell, 2016; Guyader, Ottosson, & Witell, 2017; Caspi et al., 2018; Hoppe et al., 2018).
  The glasses were connected to this laptop via a USB cable, and the SMI iViewETG software (version 2.7.1) was used for a three-point calibration and recording. The calibration was started after iViewETG’s eye model adaptation phase was completed and consisted of the user fixating the center of three specific markers in the stimulus grid (top-left corner, top-right corner, and the middle of the bottom edge). During each fixation, the experimenter clicked these locations on a live-view of the scene camera on the recording laptop. The front-facing scene camera recorded a video stream at 24 Hz with a 1280 × 960 px resolution, and each of the eye cameras recorded a video stream at 1 Hz with a resolution of 320 × 240 px. Note that the eye cameras’ frame rate refers to the recorded video provided by iViewETG and not the actual camera frame rate.
**Pupil-labs**: This setup used a setup provided by Pupil-labs, consisting of a monocular Pupil headset (both the scene and right eye cameras were Cam 1 models) and the Pupil Capture software (version 1.3.13). This is a monocular eye-tracking setup with one eye camera and two glints, although Pupil Capture does not use glint information for gaze tracking. It was chosen because the Pupil-labs platform is a popular low-cost alternative to the Tobii and SMI eye-tracking setups, and it is increasingly used in head-worn eye-tracking studies (e.g., Li, Kearney, Braithwaite, & Lin, 2018; Lappi, Rinkkala, & Pekkanen, 2017; Zhao, Salesse, Marin, Gueugnon, & Bardy, 2017). Pupil-labs explicitly claims that their software compensates for eye-tracker slippage in 3D mode,^2^ which is the default recording mode.
  The headset was connected using the provided USB cable to a HP Elite tablet running Pupil Capture set to 3D mode, which was used for a single-marker calibration and recording. The calibration consisted of (a) fixing the Pupil Calibration Marker v0.4 to the center of the stimulus grid, and (b) collecting calibration data as the participant fixates the center of this marker while rotating their head in a spiral fashion (as per Pupil-labs documentation^3^). The default Pupil Capture camera settings were used: The front-facing scene camera recorded a video stream at 30 Hz with 1280 × 720 px resolution, and the eye camera recorded a video stream at 120 Hz with a 320 × 240 px pixel resolution.
**Grip**: This setup used the aforementioned tablet and Pupil headset in combination with the open-source eye-tracking software EyeRecToo (version 2.0 commit 51a839aa). The EyeRecToo platform was used because it includes the following eye-tracking algorithms: *Grip* (Santini et al., 2019) for gaze estimation, which we chose because it is claimed to be slippage-robust, and *PuReST* (Santini et al., 2018b) for pupil tracking, which we chose because it is currently the top-performing pupil-tracking algorithm.
  During recording, gaze estimation was achieved using a bivariate polynomial regression (option *POLY_X_Y_XY_XX_YY_XYY_YXX_XXYY* in the EyeRecToo software). The recordings were later post-processed offline with Grip for gaze estimation (Santini et al., 2019), a method that was not yet available at the time of the data recording. Nevertheless, the resulting gaze estimation would be identical if Grip had been run in real-time during recording. Neither gaze-estimation method uses glint information for gaze tracking. The calibration consisted of the CalibMe (Santini et al., 2017) method, consisting of (a) fixing an ArUco marker (Garrido-Jurado et al., 2016) to the center of the stimulus grid, and (b) collecting calibration data as the participant fixated the center of this marker while rotating their head in a spiral fashion. The default EyeRecToo camera settings were used: The front-facing scene camera recorded a video stream at 30 Hz with 1280 × 720 px resolution, and the eye camera recorded a video stream at 60 Hz with a 640 × 480 px resolution.

### Procedure

Each participant was recorded on all head-worn eye-tracking setups. The participants were recorded in one of the following three orders (three participants for each order):

1. Tobii → SMI → Grip → Pupil-labs
2. Grip → Pupil-labs → Tobii → SMI
3. SMI → Grip → Pupil-labs → Tobii

For each participant, the selected order was executed twice, starting again from the beginning once the first recordings with all four eye-tracking setups were completed. This resulted in a total of 18 recordings for each eye-tracking setup (nine participants × two recordings).

Each time a participant donned each of the head-worn eye-tracking setups, the experimenter first inspected the eye camera video stream and made adjustments as necessary to guarantee that the eyes were clearly visible. The Tobii and SMI eye trackers were adjusted by selecting an appropriately sized nose pad from the nose pads provided with these systems. Additionally for the SMI, the participant was instructed to tighten the device’s headband, which was loosened only for the eye-tracker movement conditions. The Pupil headset used for the Pupil-labs and Grip eye-tracking setups was set up by adjusting the eye camera orientation and focus. The experimenter then inspected the scene camera image to make sure that the whole stimulus grid was visible and instructed the participant to adjust their head pose if required. Each eye-tracking setup was then calibrated according to their respective method (see “Apparatus”), and a check of the calibration quality was performed by asking the participant to look at the center of the four corner fixation targets on the stimulus grid. If the gaze position reported by the eye-tracking setup was significantly outside the bounds of the fixation target for any of the stimulus grid’s corners, the setup was calibrated once more. Unless it was obvious to the experienced experimenter that calibration failure was due to participant error, this second calibration was accepted and the recording procedure began.

**Fig. 3:**
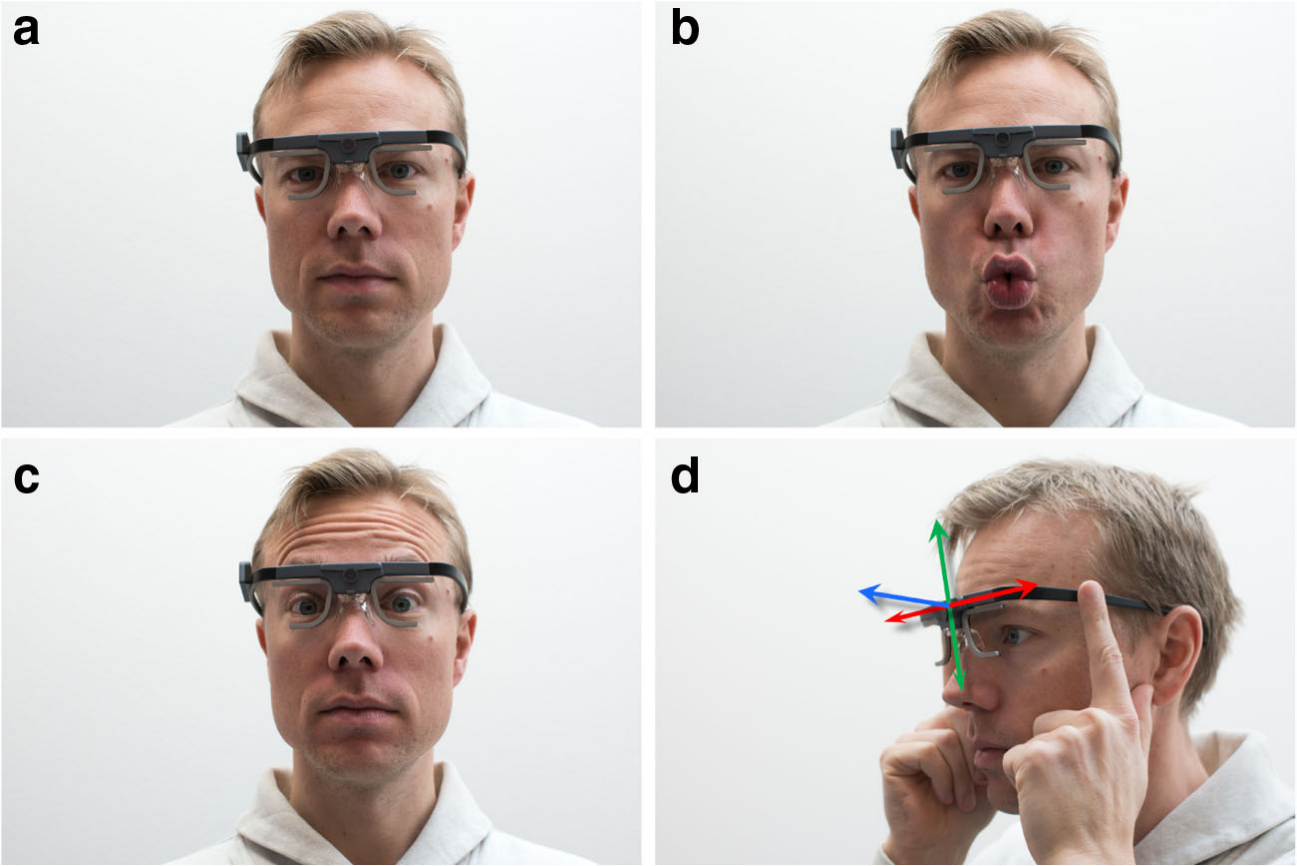
Experimental conditions. Illustration of the baseline (**a**), facial movement (**b**, **c**) and eye-tracker movements (**d**) that participants were instructed to execute. A: baseline condition. B: vowels condition, model is vocalizing a Swedish O . C: facial expression condition, model illustrates the raising of the eye brows. D: eye-tracker movement conditions, where participants held the eye tracker and moved it in the directions indicated by the arrows (horizontally [*red arrow*], vertically [*green arrow*] or toward and away from the face [*blue arrow*])

For each of the eight recordings (four head-worn eye-tracking setups × two repetitions) per participant, gaze data were recorded for the following eight conditions in the following sequence. Participants followed the following instructions:

1. **Validation** Participants were instructed to fixate the center of each of the nine fixation targets on the stimulus grid in Western reading order.
2. **No movement** Participants were instructed to fixate the center of the central gaze target for 10 s (Fig. 3a).
3. **Vowels** The experimenter voiced the Swedish vowels A (IPA phonetic: , O , U , Å , E , I , Y , Ä , Ö , and the participants were instructed to repeat them as they were voiced while maintaining fixation of the center of the central gaze target (Fig. 3b). The sequence was repeated three times. This models head-worn eye-tracker movements that may occur due to speaking.
4. **Facial expression** Participants were instructed to raise their eye brows repeatedly at a frequency of about 1 Hz while maintaining fixation of the center of the central gaze target (Fig. 3c). This models head-worn eye-tracker movements that may occur due to facial expressions.
5. **Horizontal eye-tracker movement** To model the head-worn eye-tracker shifting on the participant’s head, participants were instructed to hold the eye tracker with both hands, lift them slightly off their nose and move them horizontally for 10 s (Fig. 3d). Instructions were given to move the eye tracker for 1–2 cm at a rate of about 1 Hz. Participants were instructed to maintain fixation of the center of the central gaze target.
6. **Vertical eye-tracker movement** Same as the horizontal eye-tracker movement condition, but participants moved the eye tracker up and down repeatedly (Fig. 3d).
7. **Depth eye-tracker movement** Same as the horizontal eye-tracker movement conditions, but participants moved the eye tracker toward and away from their face repeatedly (Fig. 3d).
8. **Validation** Participants were instructed to carefully attempt to place the head-worn eye tracker back in the position where it was at the beginning of the recording, modeling participants taking off or adjusting the eye tracker. Participants were then instructed to fixate the center of each of the nine fixation targets on the stimulus grid in Western reading order.

### Data analysis

For all recordings, gaze position signals were mapped to the stimulus plane using the automated procedure described in the Appendix. As part of their pupil tracking procedure, EyeRecToo (Grip) and Pupil-labs’ Pupil Capture provide confidence values for the detected pupil. Note that these confidence values are specific to the respective software used and are not comparable between systems. Data samples with confidence values lower than 0.66 for the EyeRecToo recordings and lower than 0.6 for the Pupil-labs recordings were counted as missing data, following the recommendations from (Santini, Fuhl, & Kasneci, 2018a) and Pupil-labs,^4^ respectively. For SMI recordings, data samples were considered as missing data if the reported pupil diameter for both eyes equaled zero. For Tobii recordings, data samples were considered as missing data if the sample status indicator *“s”* was non-zero.^5^ These missing data were not included for the accuracy and precision analyses reported in this paper.

To segment the recordings into the eight conditions, author DN manually went through all recordings. Using the scene video overlaid with the recorded gaze position, and where available the eye images (Pupil-labs and Grip) and sound recordings (Tobii, SMI and Grip), he coded the start and ends of each of the conditions. Should a similar study be repeated, it is highly recommended that the experimenter provides a cue visible in the scene camera’s image to denote when conditions begin and end to make it significantly less time-consuming to determine condition starts and ends. For instance, this could be achieved by displaying unique ArUco markers at the beginning and end of each condition, which could later be automatically detected.

For the baseline, vowels, facial expression and eye-tracker movement conditions, participants were instructed to fixate the center of the central gaze target in the grid. For these conditions, the deviation between the recorded gaze point and the instructed fixation location was calculated for each sample. Per participant and eye-tracking setup, the median offset across both recordings was then determined.

We furthermore determined the amount of data loss during these conditions. The percentage data loss was determined as

1 $$  \text{Data loss} = 100 * \frac{N_{expected\_samples}-N_{valid\_samples}}{N_{expected\_samples}}, $$

where $N_{expected\_samples}$ is the number of valid data samples that was expected to be reported for a recording interval of a given duration given the nominal sampling frequency of the eye-tracking setup, and $N_{valid\_samples}$ denotes the number of samples during a recording interval for which the eye-tracking setup reported a valid gaze position.

Finally, we were interested in examining data quality across nine fixation target locations using the gaze position data recorded in the validation conditions. This necessitated labeling parts of the recorded gaze position data as fixations and then associating these fixations with one of the fixation targets. Due to highly variable data quality between and during recordings, and unknown timing of the participants’ looks to the fixation targets, part of this process had to be performed manually. As such, for the gaze position data of the validation conditions, the following procedure was used to extract accuracy, precision, and data loss measures of data quality. Note that this procedure was only used for analyzing data from the validation conditions, and was not used for post-calibration of the data analyzed in the other conditions.

1. The gaze position signal in the reference frame of the stimulus grid (see Hessels, Niehorster, Nyström, Andersson, & Hooge, 2018, for a discussion of event definitions and reference frames) was classified into episodes of saccades (periods where the gaze position changed quickly) and fixations (periods during which the gaze position remained the same) using the Hooge and Camps (2013) classification algorithm.
2. The classified fixations were then plotted on top of the raw data in the coding interface of Hooge, Niehorster, Nyström, Andersson, and Hessels (2018). Author DN manually adjusted some of the classified fixations. This was done because our goal was to quantify data quality when the participant was physically looking at one of the gaze targets regardless of drift, spikes, and intervals of data loss in the recorded data that were not due to blinks^6^ and short enough that no eye movement could have occurred. Performing this manual step ensured that episodes in the data that are normally excluded from the fixation classification by the event classification algorithm’s criteria are included in our analysis.
3. The thus-classified fixations were then manually assigned to one of the nine gaze targets on the stimulus grid using an interface that showed both a 2D view of the fixation locations on the stimulus grid’s plane, and plots of horizontal and vertical gaze position over time. This was done because there were large deviations for some or all fixation positions in some recordings, which makes it impossible to algorithmically assign classified fixations to gaze targets (e.g., using criteria such as “nearest target”). Fixations on the center gaze target before the start or after the end of the nine-point fixation sequence were not selected for further analysis.
4. To assess data quality in terms of the accuracy, precision, and data loss of the recordings, the following measures were then calculated from the selected fixations. Each measure was calculated separately for each gaze target, for each validation in each recording of each participant with each eye-tracking setup.
  - ***Deviation*** As an operationalization of accuracy, the deviation between the gaze target on the plane of the stimulus grid where a participant was instructed to look and the fixation location reported by the eye-tracking setup was used. To compute the deviation, for each selected fixation, the median distance to the gaze target it was assigned was calculated from all gaze samples in the fixation. If more than one fixation was assigned to a gaze target in the previous analysis step, the deviations calculated for these fixations were combined in an average weighted by the duration of each selected fixation.
  - ***RMS-S2S*** As an operationalization of precision, the root mean square of the sample-to-sample displacement between consecutive reported gaze positions was used. This value was calculated per gaze target in the stimulus grid for all samples that were part of the fixation(s) assigned to that gaze target.
  - ***STD*** As a further operationalization of precision that captures a different aspect of precision (Blignaut & Beelders, 2012), the standard deviation of the reported gaze position was used. The STD was calculated for each selected fixation for each gaze target. If multiple fixations were assigned to a gaze target, their standard deviations were pooled, weighted by the number of samples that comprise each fixation.
  - ***Data loss*** As for the other conditions, data loss was operationalized as the number of missing samples expressed as a percentage of the number of expected samples during a recording interval (see Eq. 1). The number of missing samples was determined by subtracting the number of samples for which the eye-tracking setup reported a valid gaze position ($N_{valid\_samples}$) from the number of samples expected for the recording interval given the nominal sampling frequency of the eye-tracking setup ($N_{expected\_samples}$). During the validation conditions, participants in total made two eye blinks. During analysis, these were not counted as data loss, as we wanted to only quantify the loss of samples when the eyes were open and visible in the eye camera image.

## Results

Videos showing example performance of each eye-tracking setup in each condition are made available in the Supplementary Material. The data and MATLAB scripts for producing the figures in this section are available at (https://github.com/dcnieho/GlassesTestCodeData).

### Raw data

**Fig. 4:**
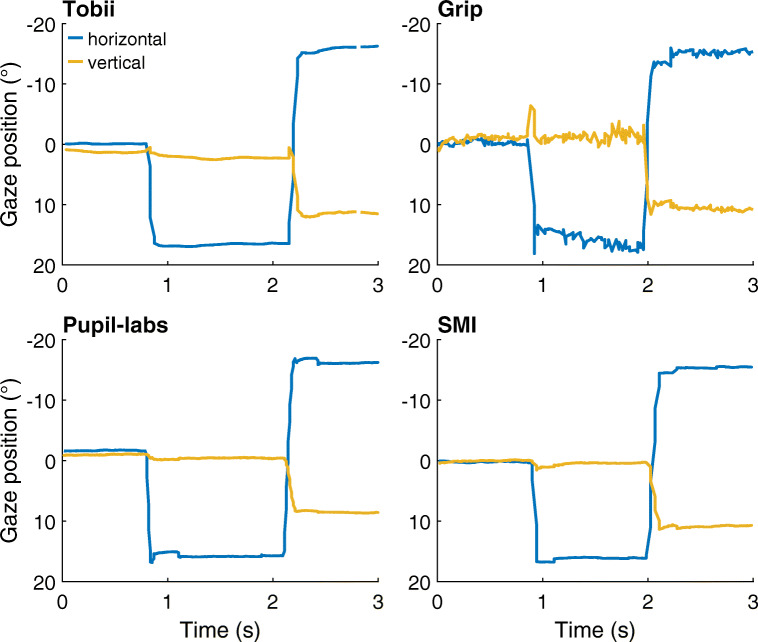
Gaze position data examples. Three seconds of representative gaze position data for each of the head-worn eye-tracking setups (*Tobii*: Tobii Glasses 2 + firmware version 1.25.3-citronkola; *Grip*: Pupil headset + EyeRecToo (Grip/PuReST); *Pupil-labs*: Pupil headset + Pupil Capture; and *SMI*: SMI ETG2 + iViewETG) during the nine-point validation condition at the start of a recording. All panels show data from the same participant. Positive is rightward for the horizontal gaze coordinate and downward for the vertical gaze coordinate. (0,0) denotes gaze toward the central gaze target

Figure 4 shows representative gaze position signals for each of the head-worn eye-tracking setups during the nine-point validation condition at the start of a recording. As can be seen in the figure, saccades are clearly visible in the signal of each eye-tracking setup as rapid changes in gaze position. However, the sample-to-sample deviations in the gaze position signal of Grip appeared to be larger than for the other eye-tracking setups. We will quantify this later as part of the data-quality assessment in Nine-point validation conditions.

### Nine-point validation conditions

In this section, data quality is analyzed for the validation tasks that participants performed at the start and end of each recording (the two validation conditions will be referred to as *validation moments* in this section). During these tasks, participants were instructed to fixate nine gaze targets laid out in a 3 × 3 grid (see Fig. 2) in Western reading order. Four data-quality variables were examined: (1) the accuracy of the measurement (the deviation in fixation location reported by the eye-tracking setup from the instructed fixation location), (2) the root mean squared sample-to-sample distance (RMS-S2S) of the gaze position signals, (3) the standard deviation (STD) of gaze position signals, (4) and the percentage of missing gaze samples (data loss).

We first examined whether data quality at the end of a recording was worse than at the start of a recording. This comparison between validation moments examined the effect on data quality of participants having moved the head-worn eye-tracking setup during the recording—as they were instructed to do in the facial and eye-tracker movement conditions—and then having put the tracker back in the starting position as well as possible before the second validation moment.

**Fig. 5:**
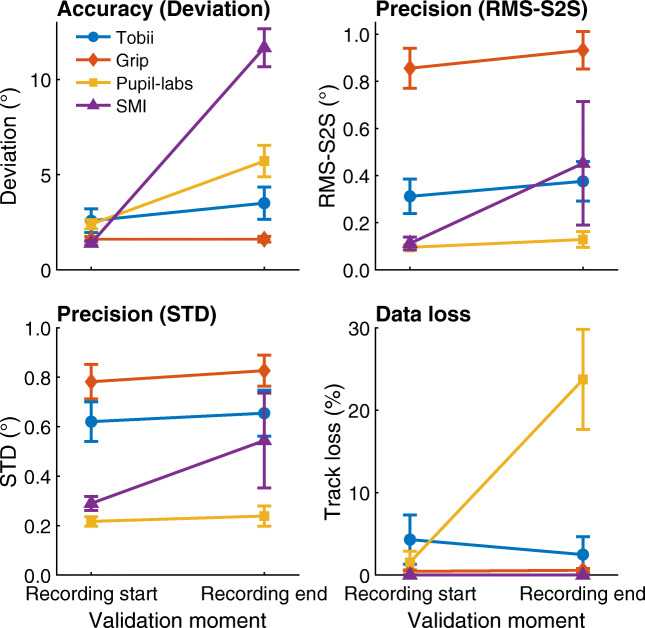
Data quality measures at the start and end of recordings. Mean deviation, RMS-S2S, STD and data loss, averaged across participants for each of the head-worn eye-tracking setups (*Tobii*: Tobii Glasses 2 + firmware version 1.25.3-citronkola; *Grip*: Pupil headset + EyeRecToo (Grip/PuReST); *Pupil-labs*: Pupil headset + Pupil Capture; and *SMI*: SMI ETG2 + iViewETG) during the validation tasks at the start and at the end of the recordings. *Error bars* denote 95% confidence intervals, indicating variation across participants

Results for the four data quality measures are reported in Fig. 5, which shows data quality averaged over the nine gaze target locations for each validation moment for each of the four eye-tracking setups. Data are presented averaged over gaze target location as no interesting interactions between gaze target location and validation moment were observed for these results.

Regarding deviation, Fig. 5 shows that while the Tobii and Grip eye-tracking setups did not show worse accuracy (larger deviation) in the validation after the eye-tracker movement tasks than before, for the Pupil-labs and especially the SMI, deviation was significantly larger during validation at recording end than at recording start.

Examining RMS-S2S and STD shows that for the Tobii, Grip, and Pupil-labs, the sample-to-sample deviation and spatial dispersion in the reported gaze data showed small differences between the two validation moments. For the SMI, mean RMS-S2S and STD were larger during validation at recording end than at recording start, but the large variability in the calculated values at recording end indicates the amount by which RMS-S2S and STD increased varied strongly over recordings. Finally, examining data loss revealed that less than 0.6 % of samples were lost for the Grip and SMI eye-tracking setups (recall that blinks were excluded from this measure when analyzing the validation tasks). The Tobii showed a nearly constant data loss of about 4%, and the Pupil-labs showed a significant data loss of 24% during validation at recording end but less than 2% during validation at recording start.

The RMS-S2S and STD panels in Fig. 5 furthermore reveal large differences in precision between the eye-tracking setups. Consistent with the gaze position signals shown in Fig. 4, the RMS sample-to-sample deviations in Grip’s gaze position signal were significantly larger than those of the other eye-tracking setups. It may furthermore be noted that the RMS-S2S value of the Tobii was more than double that of the Pupil-Labs and SMI eye-tracking setups during the first validation moment. Similarly, the STD of Grip’s and Tobii’s gaze position signals was double or more than double that of the Pupil-labs and the SMI during the first validation moment. As such, while the Tobii and Grip eye-tracking setups appeared to be robust to eye-tracker slippage (note unchanged deviation after eye-tracker repositioning), we also observed that these setups have significantly worse precision than the SMI and Pupil-labs eye-tracking setups. It could thus be that the robustness to eye-tracker slippage of the Grip and Tobii eye-tracking setups comes at the cost of worse precision in the form of higher RMS-S2S deviations and STD. The large difference in RMS-S2S between Grip and Tobii may partially arise because Grip’s gaze position signal did not undergo temporal filtering whereas we cannot exclude the possibility that Tobii applied such filters to the gaze output of their Glasses 2 eye-tracking setup.

**Fig. 6:**
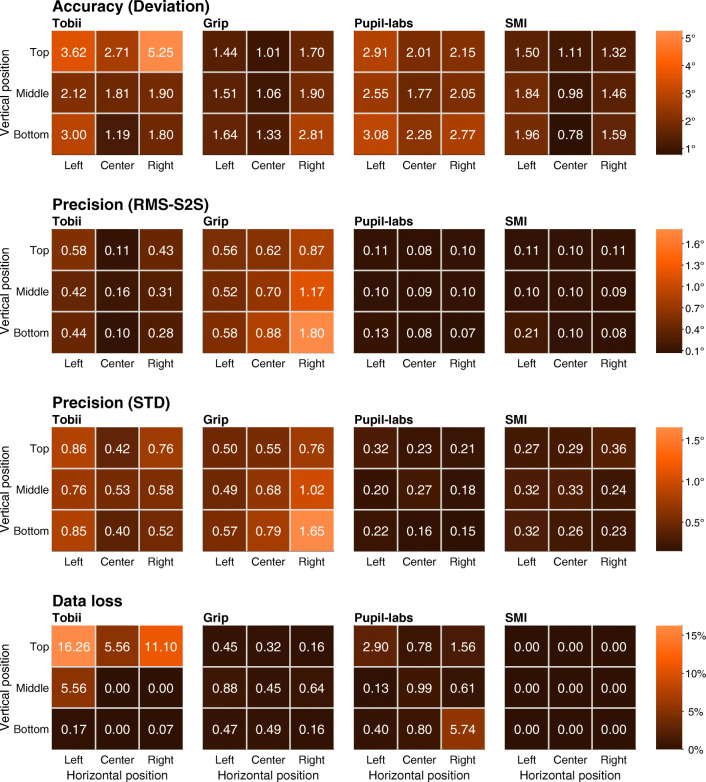
Data quality measures as a function of gaze target location. Heatmaps showing deviation (*first row*), RMS-S2S (*second row*), STD (*third row*), and data loss (*fourth row*) as a function of gaze target location for each of the head-worn eye-tracking setups (*Tobii*: Tobii Glasses 2 + firmware version 1.25.3-citronkola; *Grip*: Pupil headset + EyeRecToo (Grip/PuReST); *Pupil-labs*: Pupil headset + Pupil Capture; and *SMI*: SMI ETG2 + iViewETG), for the first validation moment at the start of a recording. In each panel, every *square* represents a gaze target location and the *color of the square* corresponds to the value determined for the given measure at that gaze target location (e.g., the top-left square in each panel indicates the data quality for the top-left gaze target). The non-central gaze targets were at horizontal eccentricities of 17^∘^ and vertical eccentricities of 11^∘^. Coloring of the squares is according to the color bar legend shown on the right of each row

Next, we examined whether data quality was dependent on gaze target location. Figure 6 shows the four data quality measures per eye-tracking setup and per instructed gaze target location for the first validation moment at the start of a recording in a heatmap format (the highest values are indicated by the brightest shades of orange, whereas the lowest values are indicated by the darkest shades). Data for the validation moment at the start of a recording is shown to provide insight into head-worn eye-tracking setup characteristics when not affected by glasses slippage.

Examining deviation, it was found that it was lowest at or close to the center gaze target location for all head-worn eye-tracking setups. All setups also showed larger deviation in reported gaze location for the four corner gaze target locations. For the Tobii, deviation especially increased for the two corner gaze target locations at the top, whereas for Grip deviations increased most for the lower-right gaze target. For the SMI and Pupil-labs eye-tracking setups, deviation increased more uniformly for all corners.

For the measures of sample-to-sample deviation (RMS-S2S) and spatial dispersion (STD) in the reported gaze position, the results varied more between setups. The Pupil-labs and SMI overall showed low RMS-S2S and STD levels that were uniform over the gaze target locations. The Tobii also showed values in the lower end of the range for RMS-S2S, but larger values for STD. Lastly, Grip showed the highest sample-to-sample deviation and spatial dispersion levels, which were lowest for the top-left gaze target location and highest for the bottom-right gaze target location, which, respectively, coincided with participant lines of sight furthest away from and closest to the eye tracker’s eye camera location. This may have arisen because Grip’s gaze estimation algorithm involves determining the 3D slant of the pupil with respect the eye camera, and variability resulting from this procedure becomes larger the more frontoparallel the pupil is to the eye camera (Santini et al., 2019).

Regarding data loss, we see that the Tobii suffered data loss predominantly in the top row of fixation target locations, and the Pupil-labs mostly in the corner fixation locations. As also reported above, the SMI eye-tracking setup did not suffer any data loss in our recordings when excluding loss due to blinks.

### Speaking and facial expressions

The main purpose of the vowels and facial expression conditions was to determine what happens when the eye tracker slips because participants speak or make facial expressions. Some speech sounds, especially when clearly articulated, involve movement of large parts of the face, and as such we may expect the head-worn eye trackers to move when some of the Swedish vowels used for this condition were pronounced. Similarly, a significant number of facial expressions involve the muscles in the nose, eye, or forehead region. Movement of any of these muscles may cause the head-worn eye tracker to slip. In the facial expressions condition, we focused on the effect of head-worn eye-tracker movement due to movement of the eyebrows.

**Fig. 7:**
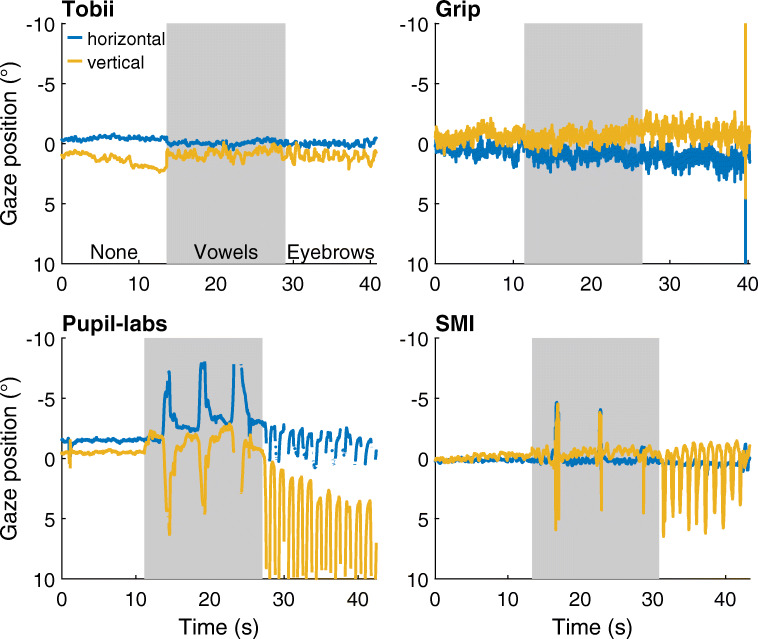
Gaze position during facial movement. Approximately 40 s of representative gaze position data during the facial movement conditions for each of the head-worn eye-tracking setups (*Tobii*: Tobii Glasses 2 + firmware version 1.25.3-citronkola; *Grip*: Pupil headset + EyeRecToo (Grip/PuReST); *Pupil-labs*: Pupil headset + Pupil Capture; and *SMI*: SMI ETG2 + iViewETG). Each of the panels is divided into three periods denoting the movements executed by the participant (labeled *None* [*white background*], *Vowels* [*gray background*] and *Eyebrows* [*white background*, referring to the facial expression condition]). All panels show data from the same participant. Positive is rightward for the horizontal gaze coordinate and downward for the vertical gaze coordinate. (0,0) denotes gaze toward central gaze target

Figure 7 shows representative gaze position signals for each head-worn eye-tracking setup while the participant fixated the center target during these facial movement conditions and a no movement baseline. As can be seen from these figures, the reported gaze position shows small deviations for the Tobii and Grip eye-tracking setups, but showed much larger deviations from the baseline gaze position in both the vowels and facial expression conditions for the SMI and Pupil-labs eye-tracking setups.

**Fig. 8:**
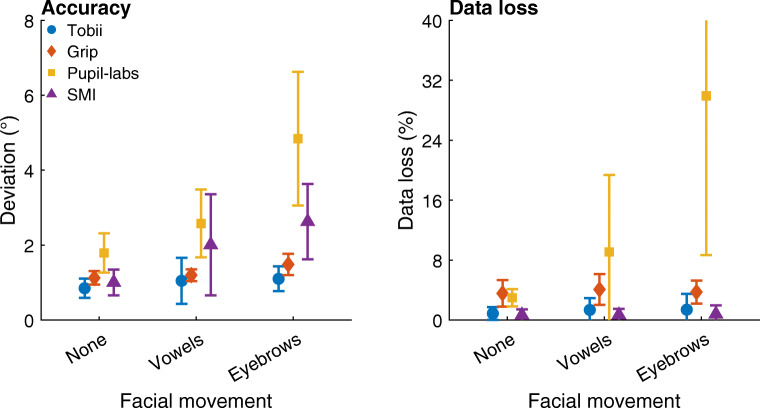
Accuracy and data loss during facial movement. *Left panel*: Mean deviation of reported gaze position averaged across participants for each head-worn eye-tracking setup (*Tobii*: Tobii Glasses 2 + firmware version 1.25.3-citronkola; *Grip*: Pupil headset + EyeRecToo (Grip/PuReST); *Pupil-labs*: Pupil headset + Pupil Capture; and *SMI*: SMI ETG2 + iViewETG) for the no movement baseline (labeled as *None*), vowels (*Vowels*), and facial expression (*Eyebrows*) facial movement conditions. *Right panel*: Mean data loss, the percentage of missing samples, averaged across participants for each eye-tracking setup. The error bars denote 95% confidence intervals, indicating variation across participants. Plotted values have been horizontally jittered for legibility

To quantify the extent to which these deviations occurred for each head-worn eye-tracking setup, the median deviations in the gaze position signal from the center gaze target during each condition were determined for each participant. The average of these medians across participants is plotted in Fig. 8 (left panel). As can be seen, for both Tobii and Grip, the deviation in gaze position during the vowels condition is nearly identical to that in the no movement condition (mean deviation vowels vs. no movement: Tobii 1.0^∘^ vs. 0.8^∘^, Grip 1.2^∘^ vs. 1.1^∘^), indicating that these eye-tracking setups are slippage-robust during this simulated speaking condition. These two head-worn eye-tracking setups showed slightly larger deviations in the facial expression condition (mean deviation Tobii: 1.1^∘^; Grip: 1.5^∘^) than in the no movement condition, suggesting that these systems are minimally affected by raising the eyebrows. In contrast, comparing the gaze deviations of the SMI and Pupil-labs eye-tracking setups in these two conditions with the no movement baseline condition indicates that the SMI and especially the Pupil-labs showed larger deviations during the vowels (mean deviation: SMI 2.0^∘^ vs. 1.0^∘^, Pupil-labs 2.6^∘^ vs. 1.8^∘^) and the facial expression conditions (mean deviation: SMI 2.6^∘^, Pupil-labs 4.8^∘^). This suggests that these two eye-tracking setups are not robust to even small amounts of glasses slippage.

In Fig. 8 (left panel), differences in gaze position output are also seen between the head-worn eye-tracking setups in the baseline no movement condition. These are due to differences in both the accuracy and precision of the eye-tracking setup. These topics have been further explored in “Nine-point validation conditions” above.

To further examine robustness of the head-worn eye-tracking setups during small movements of the eye tracker, we analyzed the percentage of missing samples. This data loss is plotted in Fig. 8 (right panel). As can be seen, data loss was low for all eye-tracking setups in all conditions except for the Pupil-labs during the vowels condition (mean across participants: 9.1%, compared to 3.0% in the no movement baseline condition) and especially the facial expression condition (30%).

### Eye-tracker movement

In the eye-tracker movement conditions, the head-worn eye tracker was displaced in a periodical fashion in front of the face while maintaining the eyes in view of the eye camera. We examined the effect of these movements on the gaze signals reported by the eye-tracking setup. These large movements mimic, for instance, when a study participant adjusts the eye tracker or is engaged in tasks involving brusque physical movement. Specifically, with these conditions, we examined how the head-worn eye-tracking setups performed during horizontal, vertical, and depth eye-tracker slippage by instructing the participants to rhythmically move the head-worn eye-tracker left–right, up–down, or toward–away in front of their face by about 1–2 cm.

**Fig. 9:**
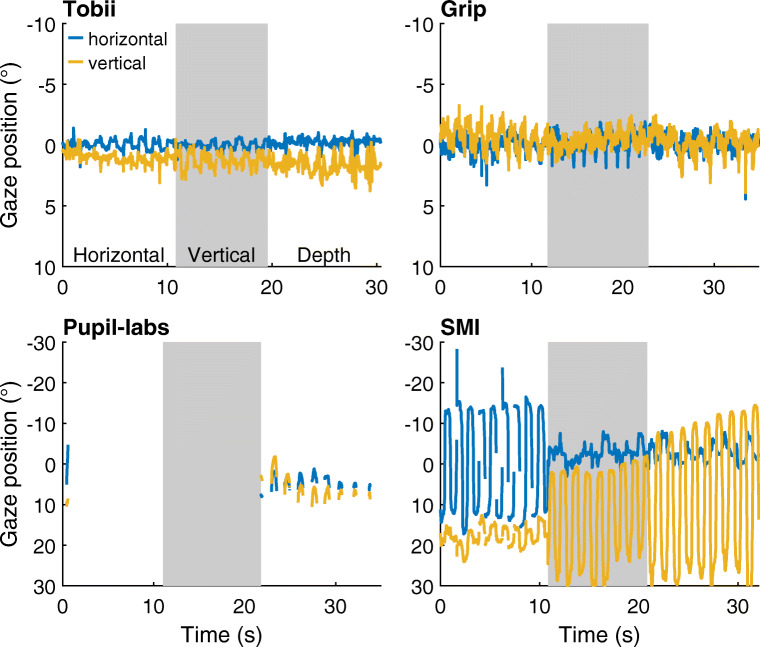
Gaze position during horizontal, vertical, and depth movement of the eye tracker. Approximately 30 s of representative gaze position data were reported during the eye-tracker movement conditions for each of the head-worn eye-tracking setups (*Tobii*: Tobii Glasses 2 + firmware version 1.25.3-citronkola; *Grip*: Pupil headset + EyeRecToo (Grip/PuReST); *Pupil-labs*: Pupil headset + Pupil Capture; and *SMI*: SMI ETG2 + iViewETG). Each of the panels is divided into three episodes denoting the eye-tracker movements with respect to the participant’s head (labeled *Horizontal* [*white background*], *Vertical* [*gray background*] and *Depth* [*white background*]). All panels show data from the same participant. Note the larger range of the ordinate for the two bottom panels. Positive is rightward for the horizontal gaze coordinate and downward for the vertical gaze coordinate. (0,0) denotes the gaze toward central gaze target

Figure 9 shows representative example gaze position signals for each head-worn eye-tracking setup in the three eye-tracker movement conditions. As can be seen from these figures, the reported gaze position remained close to the center gaze target for both the Tobii and the Grip eye-tracking setups. For the SMI, the gaze position signals showed very large deviations, while for the Pupil-labs, gaze position data were mostly lost for this participant.

**Fig. 10:**
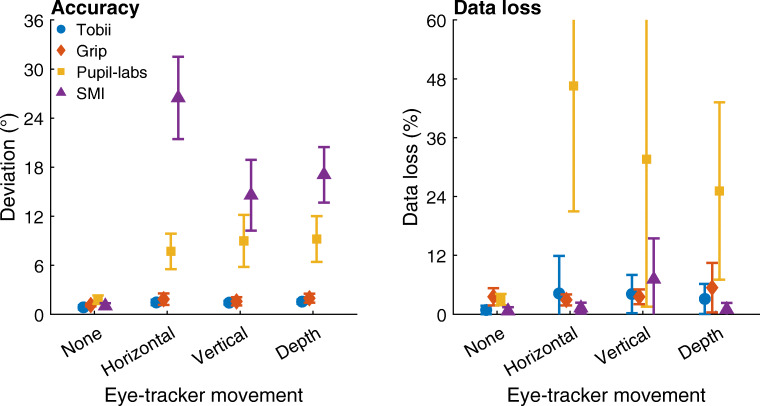
Accuracy and data loss during horizontal, vertical, and depth movement of the eye tracker. *Left panel*: Mean deviation of reported gaze position averaged across participants for each head-worn eye-tracking setup (*Tobii*: Tobii Glasses 2 + firmware version 1.25.3-citronkola; *Grip*: Pupil headset + EyeRecToo (Grip/PuReST); *Pupil-labs*: Pupil headset + Pupil Capture; and *SMI*: SMI ETG2 + iViewETG) for the no movement baseline and the three eye-tracker movement conditions. *Right panel*: Mean data loss, the percentage of missing samples, averaged across participants for each eye-tracking setup. The *error bars* denote 95% confidence intervals, indicating variation across participants. Plotted values have been horizontally jittered for legibility

To quantify the extent to which deviations in gaze position occurred for each head-worn eye-tracking setup, the median deviation in gaze position from the center gaze target during each condition was determined for each participant. The average of these medians across participants is plotted in Fig. 10 (left panel). As can be seen, both Tobii and Grip showed slightly larger gaze deviation in these three conditions than in the no movement baseline (mean deviation movements vs. no movement: Tobi 1.4–1.5^∘^ vs. 0.8^∘^, Grip 1.6–2.0^∘^ vs. 1.1^∘^), suggesting that the output of these setups is minimally affected by large eye-tracker movements. In contrast, the deviations in the gaze position signals recorded with the Pupil-labs and especially the SMI eye-tracking setups were very large (mean deviation movements vs. no movement: Pupil-labs 7.6–9.2^∘^ vs. 1.8^∘^, SMI 15–26^∘^ vs. 1.0^∘^). Consistent with the findings for the vowel and facial expression conditions, this suggests that the Pupil-labs and SMI eye-tracking setups are not robust to glasses slippage.

To further examine whether the head-worn eye-tracking setups are robust during large movements of the eye tracker, we analyzed the percentage of missing samples. This data loss is plotted in Fig. 10 (right panel). In these plots, we see that the Tobii, Grip, and SMI eye-tracking setups showed only small increases in data loss when the eye tracker was moved compared to the no movement baseline. The Pupil-labs on the other hand showed a large amount of data loss when the eye tracker moved (mean across participants: 25–47 %, compared to 3.0% in the no movement baseline condition). The observation that the Pupil-labs loses samples when the eye tracker is moved relatively far from its starting position (cf. Fig. 9) may explain the more modest increase in gaze position deviation during glasses movement in the Pupil-labs compared to the SMI.

## Discussion

In this article, we examined whether four head-worn eye-tracking setups are robust to slippage of the eye tracker with respect to the head. We examined data quality both when the head-worn eye tracker moved by small amounts because participants talked or made facial expressions and when participants moved the head-worn eye tracker for relatively larger distances in front of their face. We furthermore examined data quality directly after calibration and after the head-worn eye tracker has moved by having participants complete a nine-point validation before and after the facial movement and eye-tracker movement conditions.

### Gaze position deviation due to slippage

Our results show that the gaze position signals provided by two of the head-worn eye-tracking setups—Pupil-labs with its Pupil Capture software configured in 3D gaze estimation mode, and the SMI Glasses 2.0—were affected by movement of the eye tracker. We first compared deviation of the reported gaze position from a gaze target when the participant talked or made facial expressions to the deviation measured during a baseline condition in which the participant did not move their face. In these facial movement conditions, the Pupil-labs and SMI eye-tracking setups showed average median deviations that were 0.8–3.1^∘^ larger than the baseline no movement condition. During the eye-tracker movement conditions in which participants moved the eye tracker in front of their face, these errors became larger for both eye-tracking setups (increase of average median deviation over baseline 5.9–25^∘^). The Pupil-labs furthermore showed a large amount of data loss during the facial- and eye-tracker movement conditions (average 9.1–47%, compared to 3.0% during baseline). In contrast, the other two head-worn eye-tracking setups—the Tobii Glasses 2 and Grip running on eye images captured from the Pupil headset using the EyeRecToo software—provided gaze position estimates that showed minimal average median deviations during slippage of the eye tracker (≤ 0.4^∘^ increase over baseline in the facial movement conditions and ≤ 0.8^∘^ increase in the eye-tracker movement conditions) and low data loss (≤ 5.4*%*).

After the facial and eye-tracker movement conditions, participants were instructed to place the eye tracker back on their head in its initial position. Participants were then instructed to fixate a series of gaze targets and the gaze position signals obtained during this validation condition were compared to those obtained when performing the same validation at the start of each recording. Deviations in gaze position were very similar between these two validation moments for the Tobii and Grip eye-tracking setups, whereas for the Pupil-labs and especially the SMI a marked increase in deviation was found between the two validation moments. Data loss at the second validation moment was higher than at the first only for the Pupil-labs. This finding complements the observation that the Pupil-labs and SMI were not robust when the eye tracker undergoes movement during the facial and eye-tracker movement conditions, showing that these eye-tracking setups remained inaccurate even thereafter when the eye tracker was no longer moving.

The above findings strongly indicate that knowledge of how one’s head-worn eye-tracking setup performs during slippage is paramount when designing a study that makes use of head-worn eye-tracking setups. For slippage-robust eye-tracking setups, using measurement protocols that include validation tasks at the start and the end of each recording, or at regular intervals during the recording, should be sufficient to reveal data-quality issues that may have arisen due to eye-tracker slippage during the recording. However, for slippage-sensitive eye-tracking setups, such validation tests do not suffice: As shown in this study, unavoidable participant behaviors that implicitly (e.g., speech and facial expressions) or explicitly (e.g., eye-tracker adjustments) cause the eye tracker to slip can introduce large and volatile momentary gaze position deviations. Such momentary gaze position deviations would not be picked up at all by validation tests. Slippage not only leads to large deviations in recorded gaze position, but it also alters the gaze signal’s dynamics. For instance, movements in the gaze position signal that are caused by eye-tracker slippage instead of eyeball rotation could lead eye-movement-classification algorithms to label episodes of the gaze position signal as, e.g., saccades or smooth pursuit when one would want these to be labeled as fixations.

Slippage-induced gaze deviations and altered gaze signal dynamics can further undermine the outcomes of studies when the frequency or magnitude of eye-tracker slippage may differ between experimental conditions or between participant groups. Examples where this may occur are studies of (a) dyadic interaction where one person speaks and the other only listens, (b) examinations of gaze behavior of groups of people that differ in facial expressiveness or communicative skills (e.g., autism spectrum disorder, ASD), (c) comparisons of gaze behavior between attentive and distracted students, or (d) comparisons between healthy controls and attention-deficit hyperactivity disorder (ADHD) patients or clinical groups such as Alzheimer and Parkinson patients. In such comparisons, differences in facial and eye-tracker movement are likely to occur. The consequent differences in eye-tracker slippage may induce gaze signal differences that are confounded with the putative differences in gaze-behavior that are the object of study. As such, unless great care is taken in equipment choice, equipment testing, and study design, a study making such comparisons may end up reporting results that reflect flaws in the recording equipment rather than differences in gaze behavior.

An example of a study whose results may be compromised by slippage-induced gaze position deviations is Freeth and Bugembe (2018). In this study, a slippage-sensitive eye-tracking setup (SMI Glasses) was used to compare gaze to small areas of interest (parts of faces) between individuals with ASD and typically developing controls. Not only is it well known that (non-)verbal communication is impaired in ASD (American Psychiatric Association, 2013), but there are also reports of differences in facial and vocal expressiveness between individuals with ASD and controls (Faso, Sasson, & Pinkham, 2015; Grossman, Edelson, & Tager-Flusberg, 2013). Consequently, it is unclear what gaze deviations the data of Freeth and Bugembe (2018) contained and how these differed between groups, and thus whether their data are of sufficient quality to support their conclusions.

We therefore urge researchers to interpret gaze behavior from head-worn eye-tracking setups with caution, particularly if a slippage-sensitive eye-tracking setup is used. Moreover, as demonstrated in our study, even claims of slippage compensation for some eye-tracking setups might be overstated—e.g., the Pupil-labs. Therefore, we additionally recommend researchers to conduct their own tests with the eye-tracking setup and their target population to check the manufacturers’ claims and become aware of additional data-quality issues that may arise from the study participants’ physiology or behavior. Only when the results of these tests are fed into a study’s design can it be ascertained that sufficient data quality is achieved, yielding recordings from which valid conclusions can be drawn.

### Further points when choosing a head-worn eye-tracking setup

The data from the validation conditions furthermore allowed assessing precision, another aspect of data quality, of the four eye-tracking setups. Precision was assessed by means of the sample-to-sample deviation (RMS-S2S) and the standard deviation (STD) of the gaze position signal during fixation of the gaze targets. These analyses revealed that while the Tobii and Grip eye-tracking setups are robust to eye-tracker slippage, the gaze position signals recorded with these setups also showed lower precision compared to the other two eye-tracking setups. During validation at the start of a recording, the Tobii setup and especially the Grip eye-tracking setup showed markedly higher RMS-S2S and STD than the Pupil-labs and the SMI. RMS-S2S and STD remained at the same level when assessed again at the end of a recording session for the Tobii, Grip, and Pupil-labs eye-tracking setups, but increased for the SMI, reaching levels similar to those of the Tobii.

Taking the above data-quality assessments together, there is an important trade-off when deciding which head-worn eye-tracking setup to use. Among the selection of eye-tracking setups examined in this article, users who wish to optimize for robustness to eye-tracker slippage in the gaze position signals would likely prefer the Tobii or Grip. While users who wish to optimize for precision may prefer the SMI and Pupil-labs eye-tracking setups, usage of these setups comes with the important large limitation that slippage of the eye tracker must be prevented, which severely limits their use cases. Here it is worth noting that advanced head-mounted eye-tracking users may wish to build their own eye-tracking setups with different properties, such as slippage-robustness, good precision, or other desired characteristics, by changing the software component of their eye-tracking setup. This possibility is afforded by open hardware platforms that provide unrestricted access to the cameras in the headset, such as those offered by Pupil-labs, Dikablis, and Positive Science, in combination with open-source and extensible eye-tracking software like EyeRecToo.

The results of the validation conditions furthermore indicated that for the Tobii, accuracy was worse (deviations were larger) and data loss higher at the three top gaze targets, whereas other systems did not show such a decrease in data quality for upward gaze directions. As the scene camera of the Tobii eye tracker is also angled further downward than the other systems, this suggests that the design of the Tobii eye-tracking setup is optimized for recording gaze during tasks that are performed below eye height, such as on a table-top or in a supermarket. The worse data quality when looking up means that the Tobii eye-tracking setup is less suitable for use during tasks that elicit gaze at eye height and above, such as face-to-face interaction tasks, or use when the chin is lowered, such as during gun shooting tasks.

**Fig. 11:**
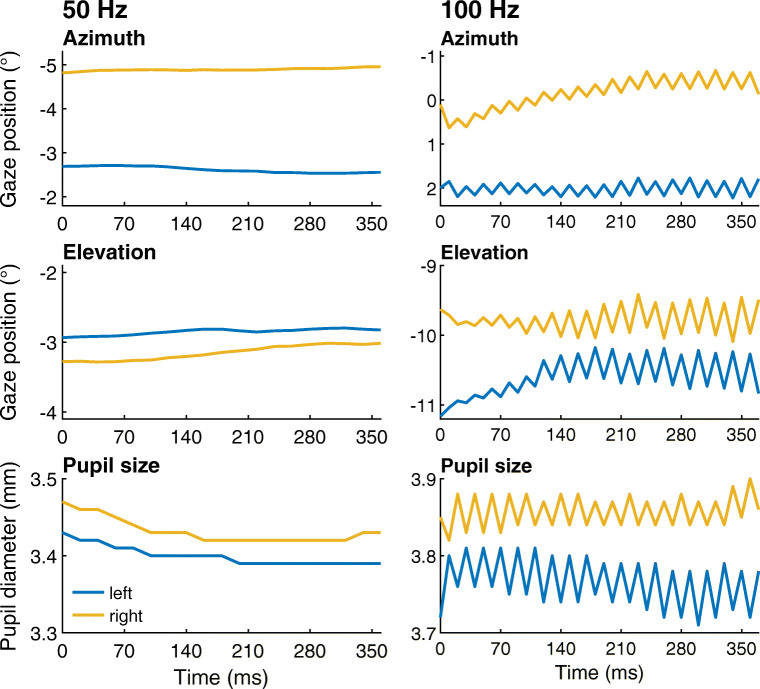
Data from Tobii Glasses 2 at 50 Hz and 100 Hz. Approximately 360-ms segments of representative gaze orientation and pupil size data recorded at 50 Hz (*left panel*) and 100 Hz (*right panel*) with the Tobii Glasses 2 while the participant fixated points on the stimulus grid. The axis ranges for both top panels, both middle panels, and both bottom panels are the same. The gaze vectors reported by the eye-tracking setup for each eye were decomposed into Fick angles (Fick, 1854; Haslwanter, 1995) and plotted over time along with pupil diameter. Positive is rightward for the azimuth (horizontal) gaze coordinate and downward for the elevation (vertical) gaze coordinate

The Tobii Glasses 2 eye tracker has a 100-Hz mode as an upgrade option. For this study, we initially planned to record using a Tobii eye-tracking setup at 100 Hz. However, we noticed that when recording at this sampling frequency, the gaze position signal frequently contained a large saw-tooth pattern (see Fig. 11, right panel) that was not found in data recorded at 50 Hz (Fig. 11, left panel). To us, it seems unlikely that this rapidly oscillating saw-tooth pattern originates from human eye movements. The sample-to-sample distance in the saw-tooth depicted in Fig. 11 ranges from about a third of a degree to about a degree, which is representative for the magnitude we observed in a few recordings. Segments with much larger modulations are also sometimes found, often for more extreme gaze angles. The saw-tooth artifact in the gaze position signals might significantly complicate analysis of the eye-tracking data, such as saccade classification. Given that the saw-tooth artifact is present in the 100-Hz mode of the Tobii Glasses 2 while we have not observed it in data recorded with the Tobii’s 50-Hz mode, we strongly recommend that researchers only use the 50-Hz mode.

### Conclusions

The results of our tests have shown that the data quality of the gaze position signal provided by head-worn eye-tracking setups can be markedly lower when the eye tracker moves with respect to the participant’s eyes. Even small movements of the eye tracker that readily occur during speaking and when making facial expressions can lead to large deviations in the gaze position signal (0.8–3.1^∘^ increase in median deviation over baseline). Our results also showed that the gaze position signals of two eye-tracking setups, the Tobii Glasses 2 at 50 Hz and Grip as implemented in the EyeRecToo software, were affected only little by eye-tracker slippage, even during larger movements of the eye tracker in front of the participant’s face. These findings underscore that it is important for researchers to ascertain whether the gaze position signals provided by their eye-tracking setup are affected by slippage during their recordings. Researchers could partially achieve this by including a validation at the end of recordings, and possibly also validation moments during recordings. Nonetheless, one should be aware that in between these validations, slippage-induced gaze deviations due to even minute eye-tracker movements might still occur and go undetected, in particular for slippage-sensitive eye-tracking setups.

It is therefore furthermore recommended for researchers to perform tests inspired by those reported in this article to become familiar with their eye-tracking setup’s performance and operating characteristics and to learn how the effects of slippage present themselves in the gaze position signal of their eye-tracking setup. While the results reported here are likely predictive of the performance of the tested eye-tracking setups when used in other experiments, our results should not be used as a best buy guide. Instead, our results necessitate the conclusion that researchers using head-worn eye-trackers must adopt a practice of testing their equipment. Ideally, these tests are performed briefly at the start of each recording because slippage-induced artifacts are different for each participant. This way, researchers are able to design their experiment and analyze their eye-tracking data in accordance with the data quality they may expect. While some may find motivation in our results to build custom eye-tracking setups, also the vast majority of eye-tracking researchers who stick to off-the-shelf eye-tracking setups are not freed from the responsibility to know the limitations imposed by the data quality of their recordings.

Finally, as is the case for tower-mounted and remote eye tracking, it is imperative that researchers do not simply repeat manufacturer specifications (Hessels et al., 2015; Niehorster et al., 2018), but instead report data-quality measures obtained from validations performed at the start and at the end of recordings (see Santini et al., 2018, for an example) in their articles. When designing their study, researchers should keep in mind the limitations placed on data interpretation by the accuracy of the gaze position signal of their eye-tracking setup as revealed by these validations, as well as by tests of eye-tracking setup performance during slippage.

### Electronic supplementary material

Below is the link to the electronic supplementary material.

(ZIP 35.2 KB)

(MP4 121 MB)

(MP4 79.5 MB)

(MP4 67.8 MB)

(MP4 129 MB)

## Figures and Tables

**Fig. 12:**
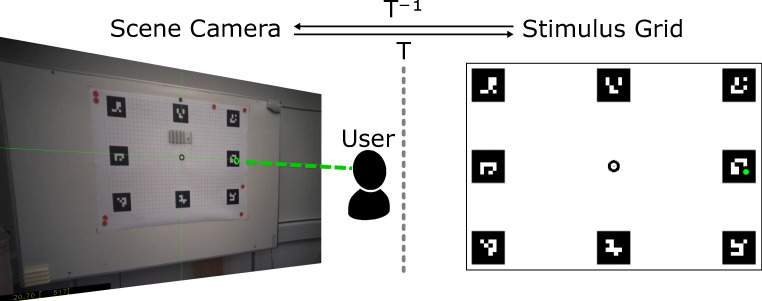
Mapping gaze position from the scene camera to the plane of the stimulus grid. By automatically detecting the ArUco markers at the border of the stimulus grid in scene camera images, we determined the transformation (*T*) that mapped points in the scene camera image to the stimulus grid by estimating the homography from detected marker positions to reference positions on the stimulus grid

## Appendix: Method for automated mapping of gaze to stimulus plane

Each eye-tracking setup produced data in their own format. To enable processing the data from the different setups with a single processing method, we first converted the recordings of each eye-tracking setup to one standardized format. This standard data format consisted of (1) a scene video, (2) a file containing frame timestamps for the scene video, and (3) a tab-separated file containing gaze points in the head-referenced coordinate system of the scene video, their associated timestamps, and the associated frame index in the scene video. This was achieved for the Tobii, SMI, and Pupil-labs based on the scripts provided by MacInnes et al., (2018) with small modifications to match our setups. For EyeRecToo recordings, we wrote a similar script. The remainder of the appendix refers to this standard file format.

For each frame in the scene video, we determined a transformation function (*T*) that mapped the gaze coordinates in the scene video to the stimulus grid as illustrated in Fig. 12. This was achieved as follows:

1. If necessary, the frame was first undistorted through precalculated intrinsic camera parameters obtained through a typical camera calibration procedure. This was the case for the Pupil-labs and Grip eye-tracking setups using the Pupil headset; we used the same intrinsic parameters for both. For the Tobii and SMI eye-tracking setups, the scene videos did not present obvious visible lens distortions and, thus, no undistortion was applied.
2. Afterwards, the eight ArUco markers at the border of the stimulus grid were automatically detected in the (undistorted) frame using OpenCV (version 3.4.3). The transformation function (*T*) was then estimated as the homography which maps the detected marker position to their reference position in the stimulus grid.
3. The correctness of this transformation was verified by applying its inverse (*T*^− 1^) to the center point of the stimulus grid plane (as well as distorting the transformed point if necessary) and visually inspecting that the resulting point matches the center of the grid in the (distorted) scene video.

Afterwards, each gaze point in the common file format can be mapped from the scene image to the stimulus grid by (1) applying the undistort function if necessary, and (2) applying the associated frame’s transformation to the (undistorted) gaze point.

## Footnotes

1 https://www.tobiipro.com/product-listing/tobii-pro-glasses-2/, accessed 2019-03-07.

2 https://pupil-labs.com/pupil/, accessed 2019-03-07.

3 https://docs.pupil-labs.com/#calibration-methods, accessed 2019-03-07 – see also the paper that introduces and evaluates this type of calibration, Santini, Fuhl, and Kasneci (2017).

4 https://github.com/pupil-labs/pupil-docs/blob/master/user-docs/data-format.md, accessed 2019-03-05.

5 According to the *Tobii Pro Glasses 2 API Developer’s Guide*: “any non-zero value indicates some kind of problem with the data”.

6 Although participants were not given instructions to avoid blinking, only two clear blinks were detected in the gaze position signal and excluded from analysis by author DN.
