# Supplementary figures and images for: The impact of slippage on the data quality of head-worn eye trackers
Source: Behav Res Methods. 2020 Jan 2;52(3):1140–60. doi: 10.3758/s13428-019-01307-0 (PMC7280360; doi:10.3758/s13428-019-01307-0)

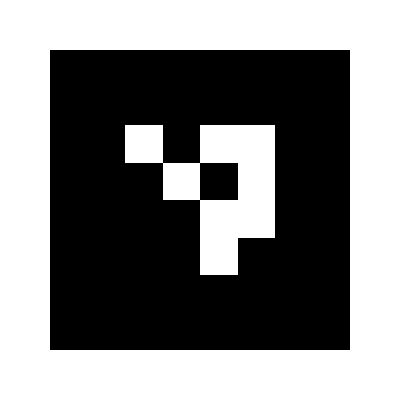

Supplement: Supplementary file 1 — (ZIP 35.2 KB) [file 13428_2019_1307_MOESM1_ESM.zip › stimulus_poster/all-markers/0.png]

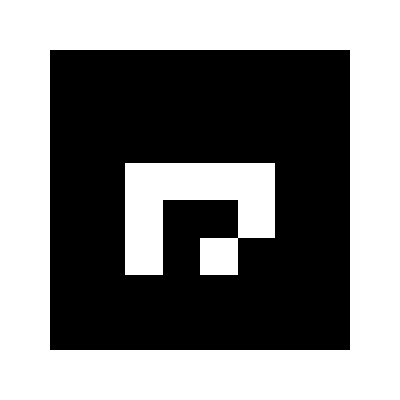

Supplement: Supplementary file 1 — (ZIP 35.2 KB) [file 13428_2019_1307_MOESM1_ESM.zip › stimulus_poster/all-markers/1.png]

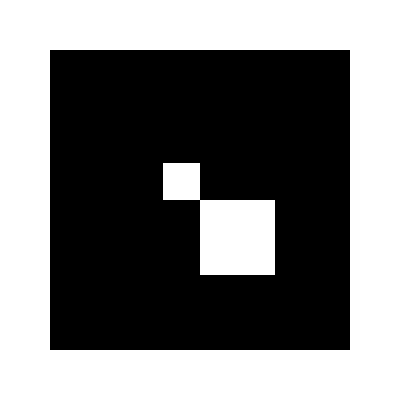

Supplement: Supplementary file 1 — (ZIP 35.2 KB) [file 13428_2019_1307_MOESM1_ESM.zip › stimulus_poster/all-markers/128.png]

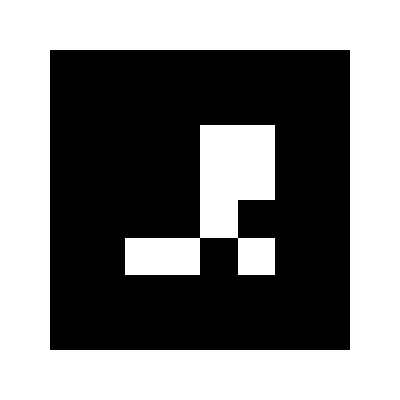

Supplement: Supplementary file 1 — (ZIP 35.2 KB) [file 13428_2019_1307_MOESM1_ESM.zip › stimulus_poster/all-markers/2.png]

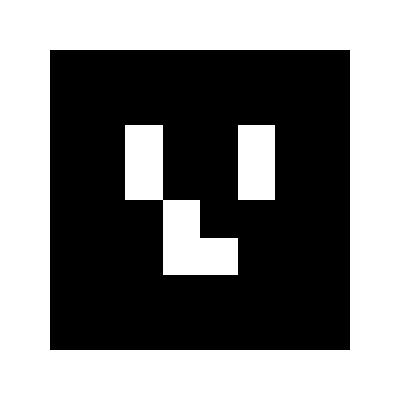

Supplement: Supplementary file 1 — (ZIP 35.2 KB) [file 13428_2019_1307_MOESM1_ESM.zip › stimulus_poster/all-markers/3.png]

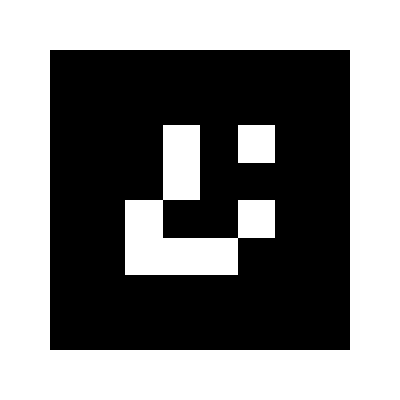

Supplement: Supplementary file 1 — (ZIP 35.2 KB) [file 13428_2019_1307_MOESM1_ESM.zip › stimulus_poster/all-markers/4.png]

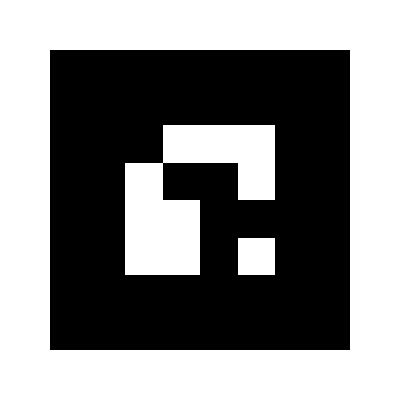

Supplement: Supplementary file 1 — (ZIP 35.2 KB) [file 13428_2019_1307_MOESM1_ESM.zip › stimulus_poster/all-markers/5.png]

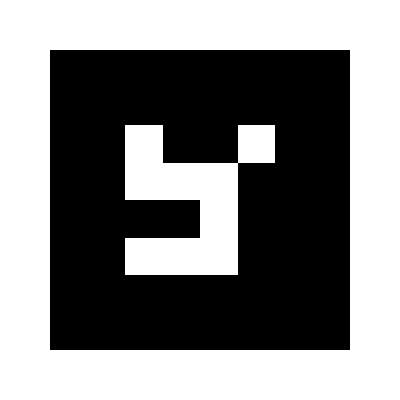

Supplement: Supplementary file 1 — (ZIP 35.2 KB) [file 13428_2019_1307_MOESM1_ESM.zip › stimulus_poster/all-markers/6.png]

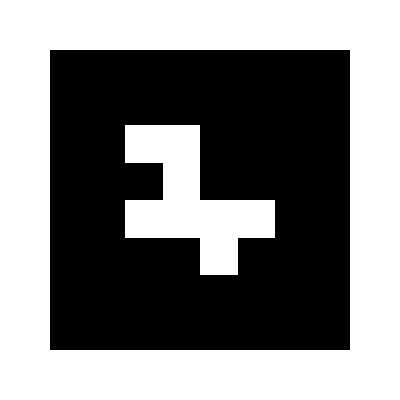

Supplement: Supplementary file 1 — (ZIP 35.2 KB) [file 13428_2019_1307_MOESM1_ESM.zip › stimulus_poster/all-markers/7.png]

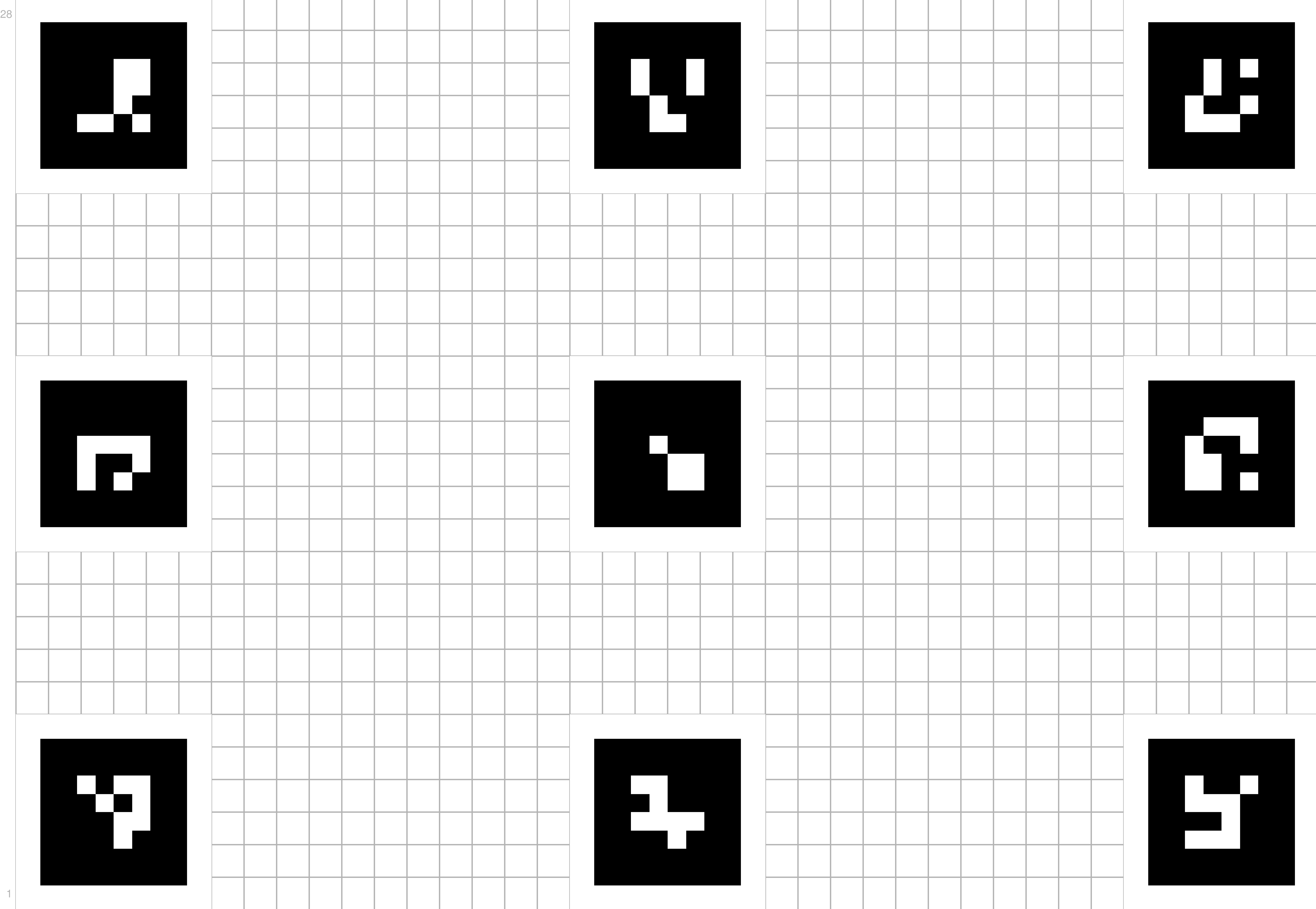

28

1

Supplement: Supplementary file 1 — (ZIP 35.2 KB) [file 13428_2019_1307_MOESM1_ESM.zip › stimulus_poster/poster_EyeRecToo.pdf]

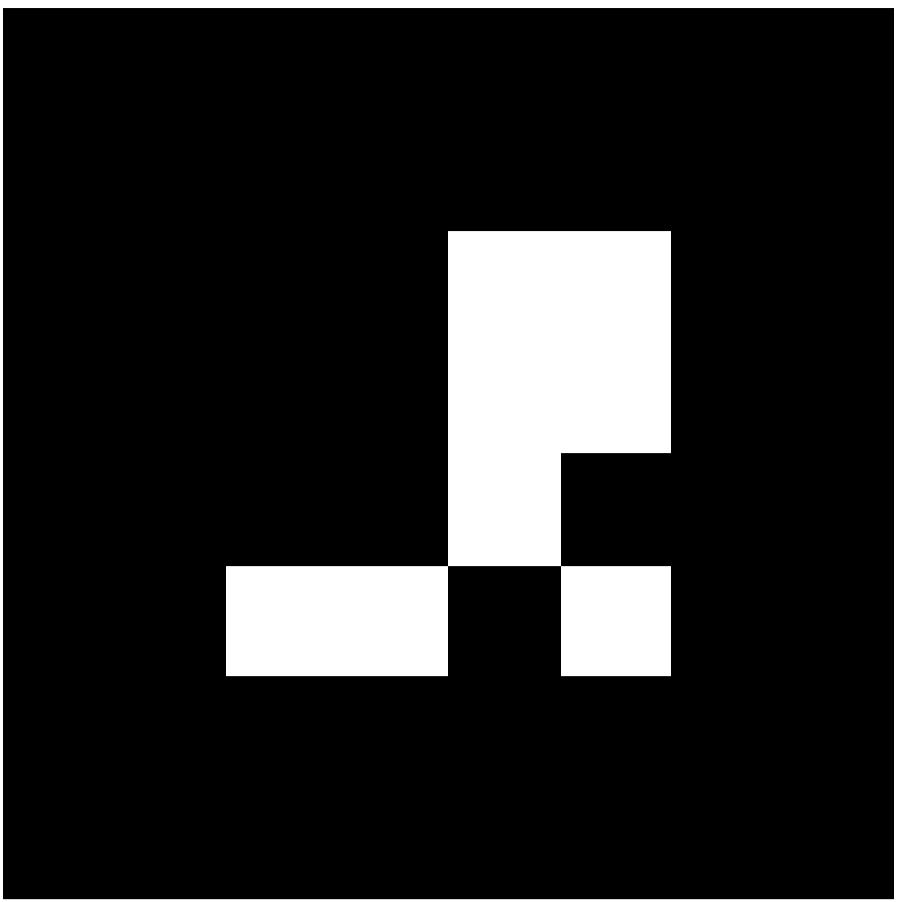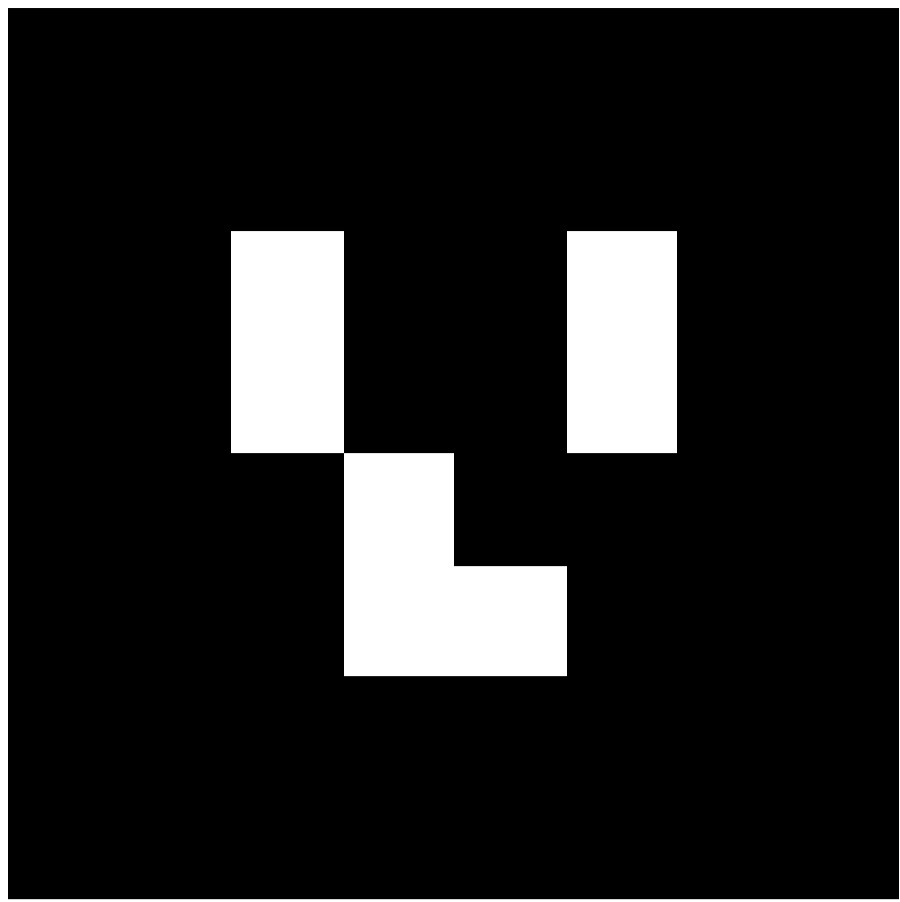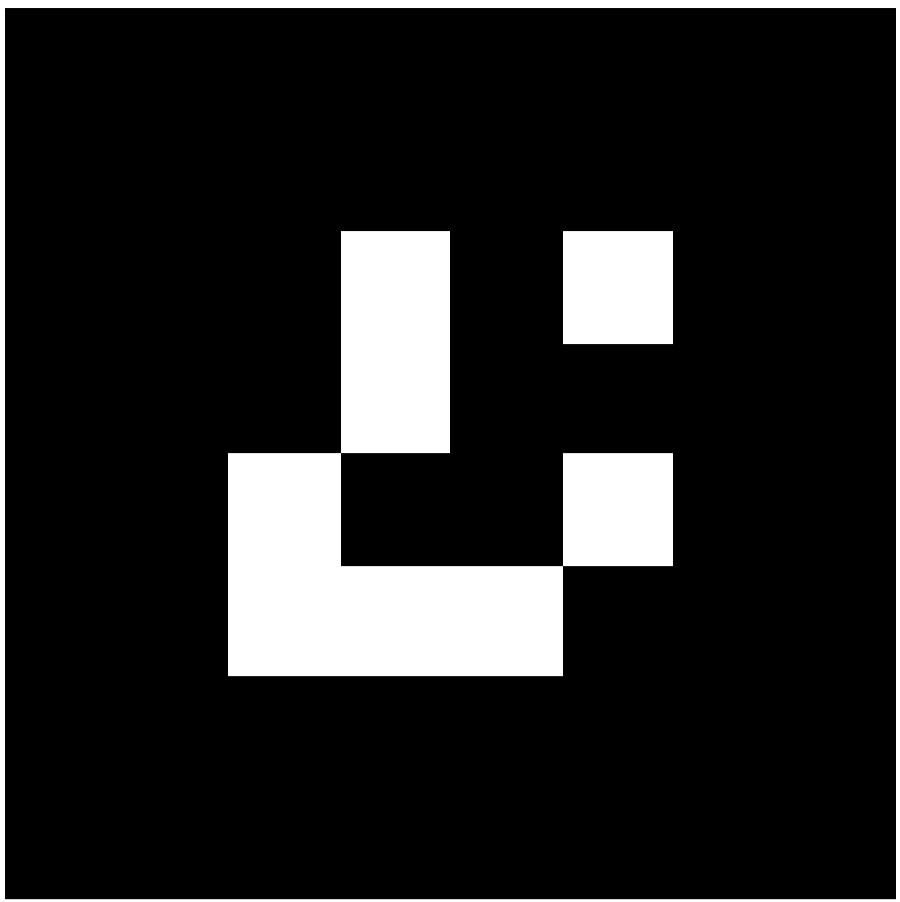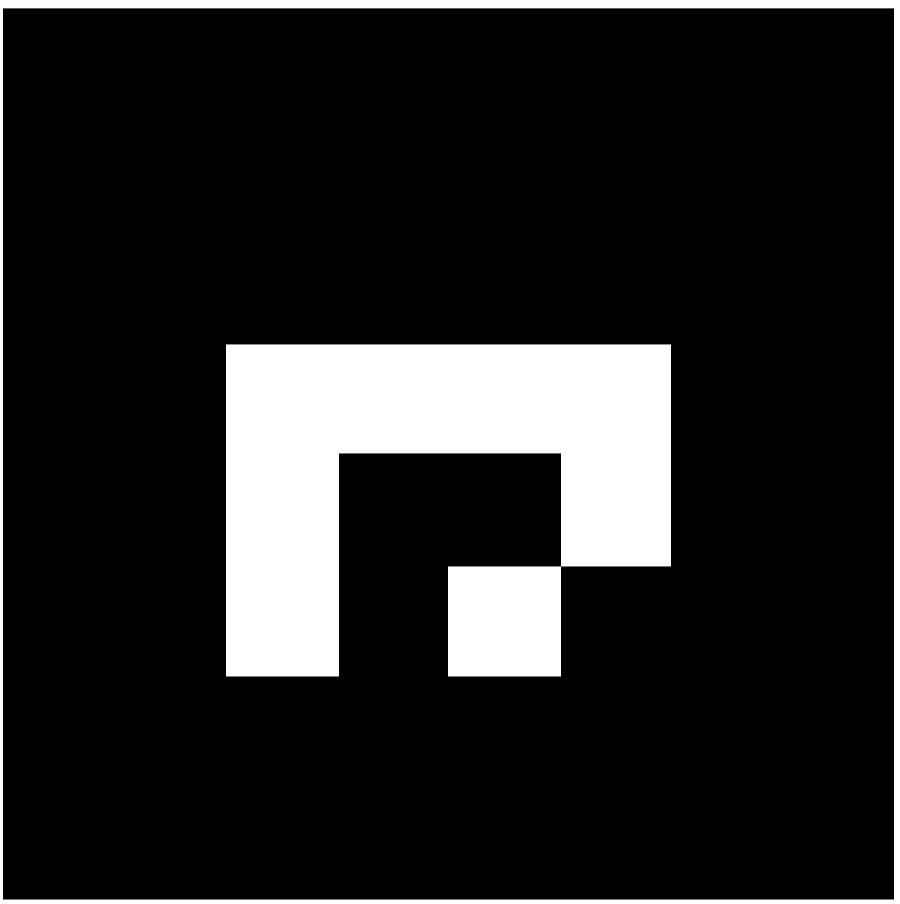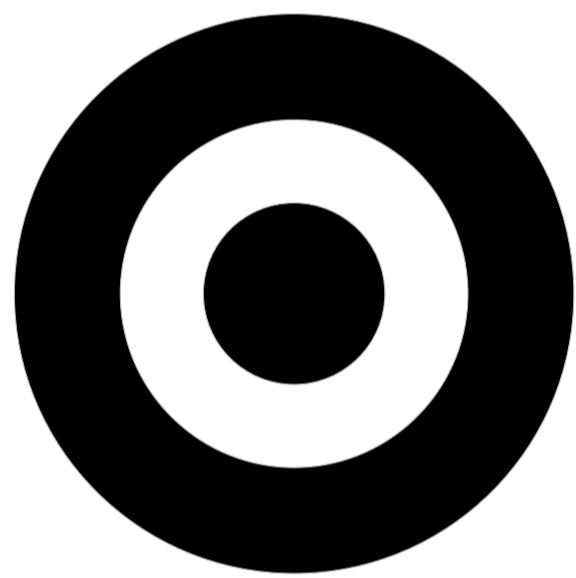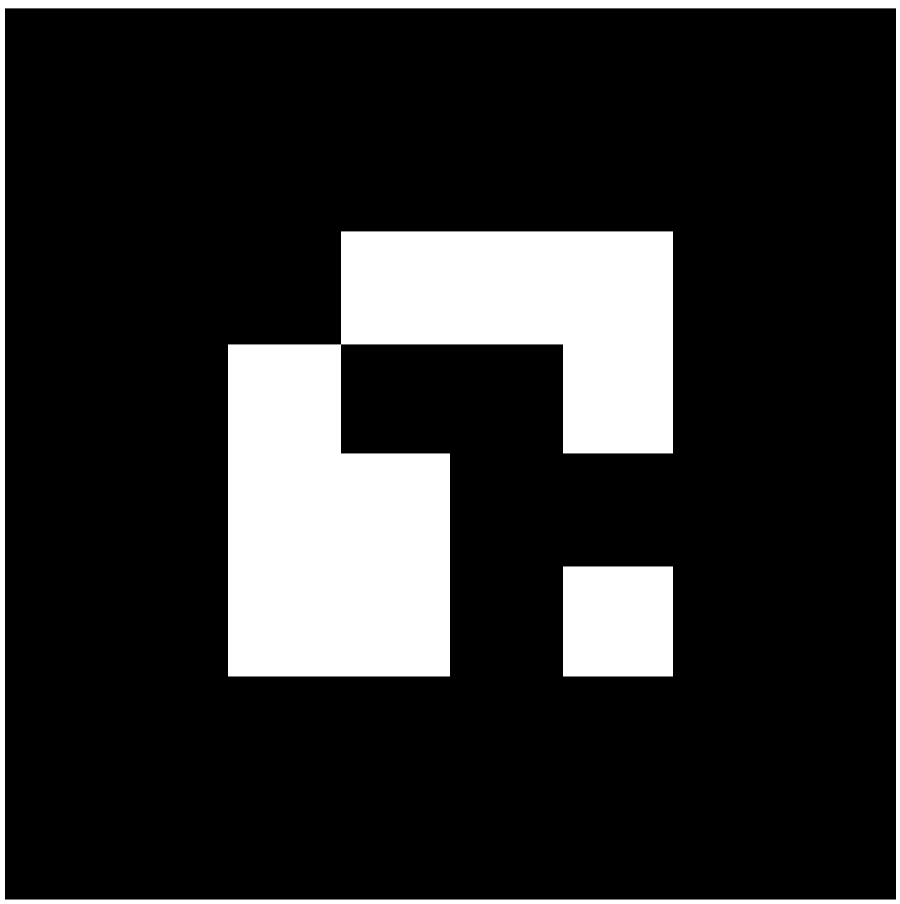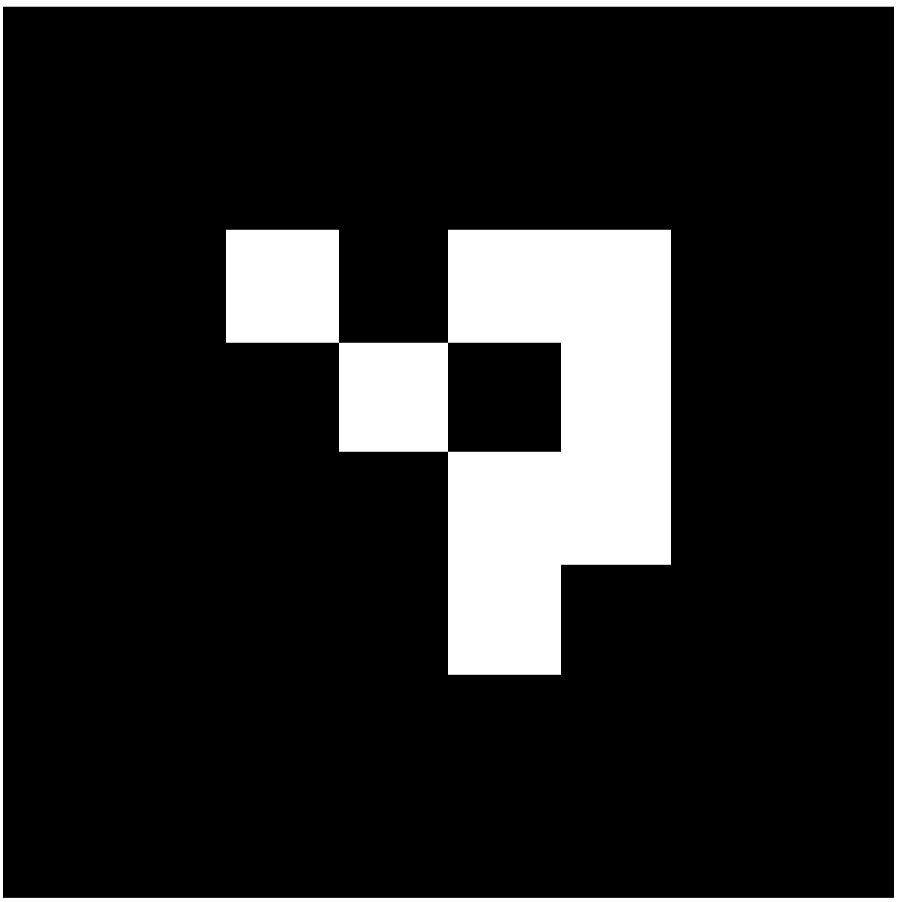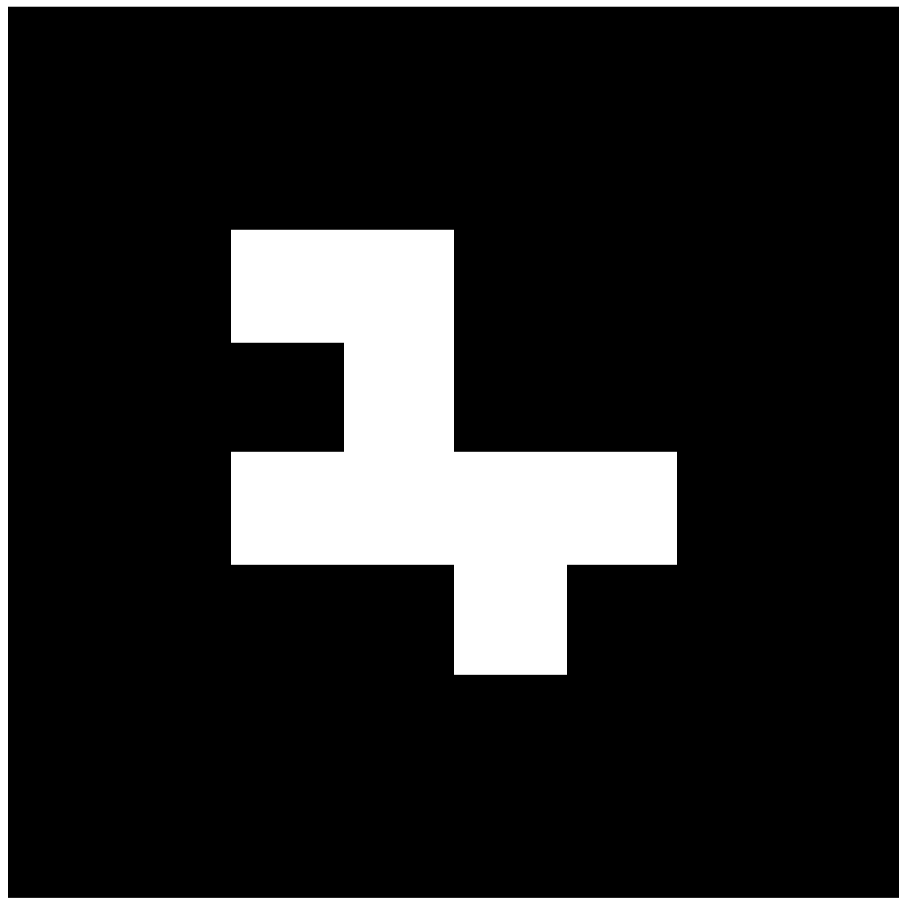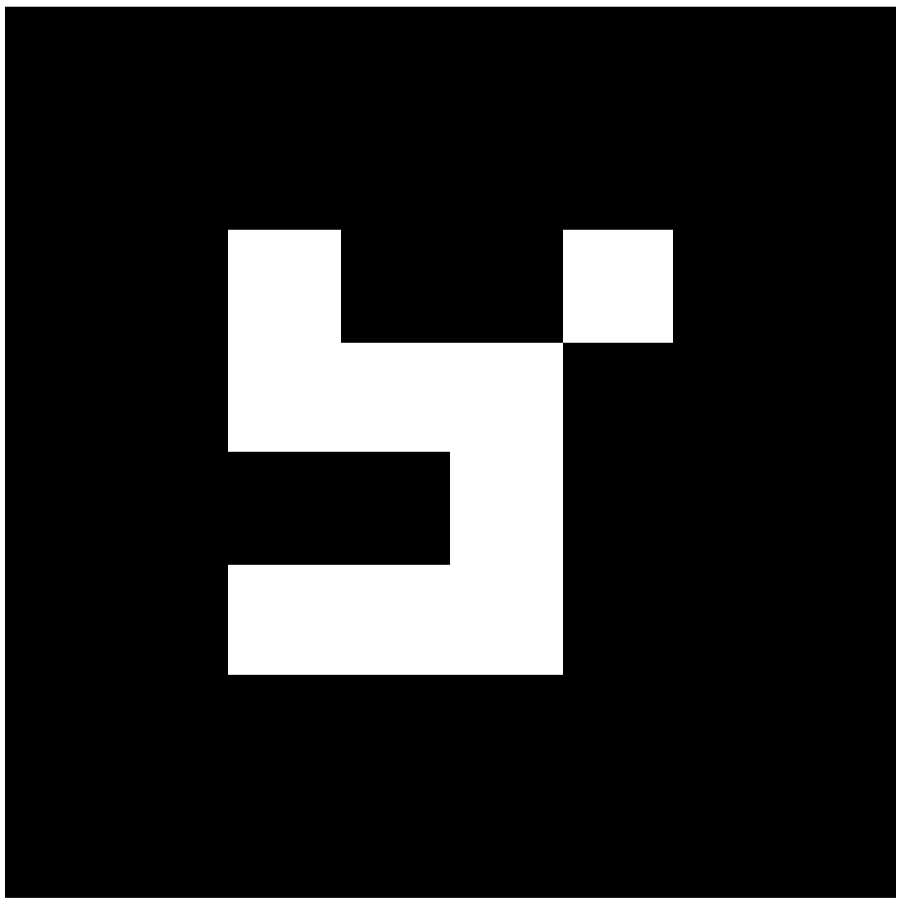

Supplement: Supplementary file 1 — (ZIP 35.2 KB) [file 13428_2019_1307_MOESM1_ESM.zip › stimulus_poster/poster_PupilLabs.pdf]

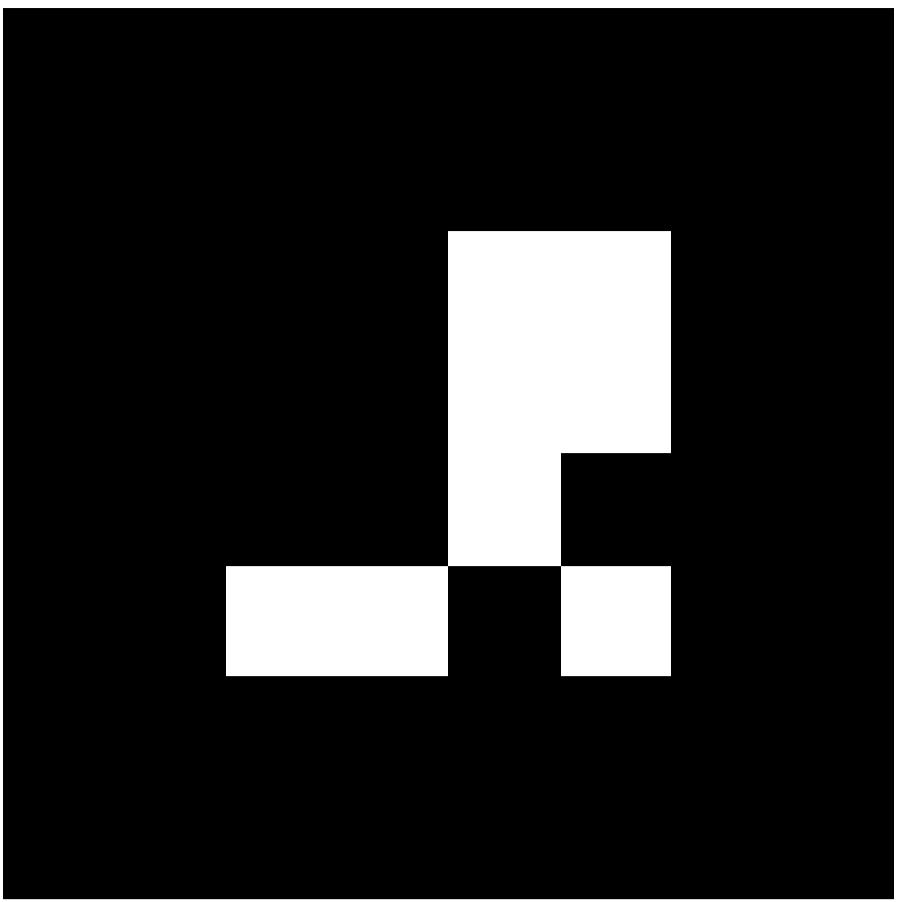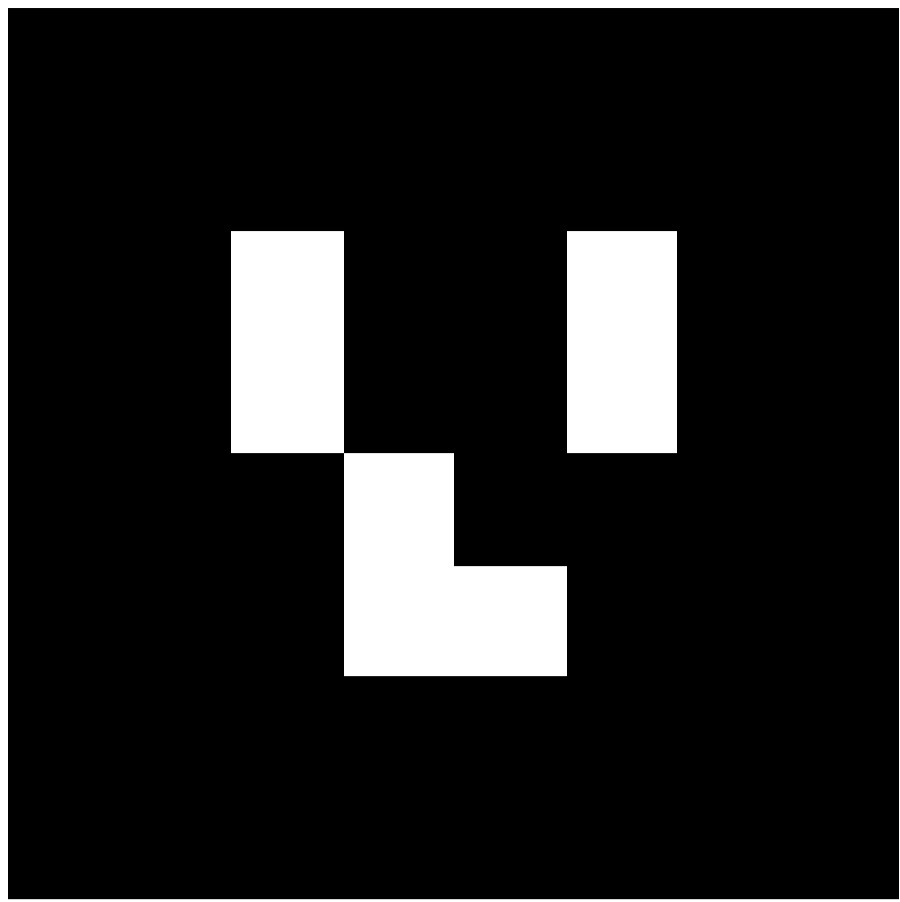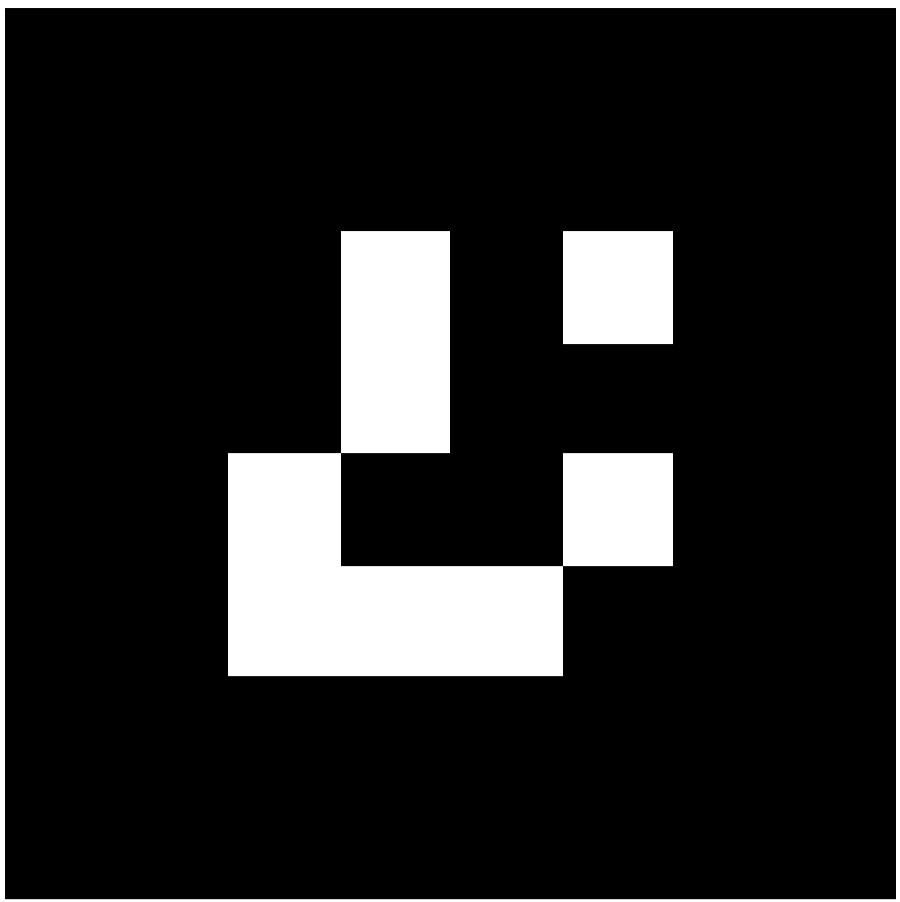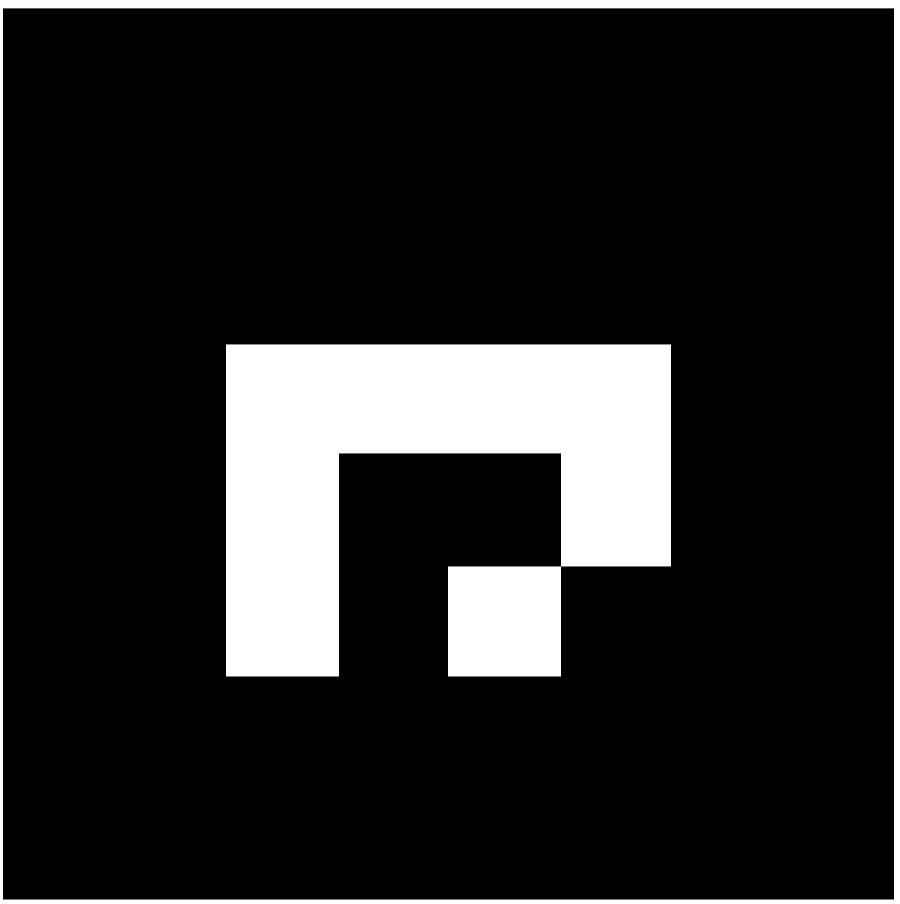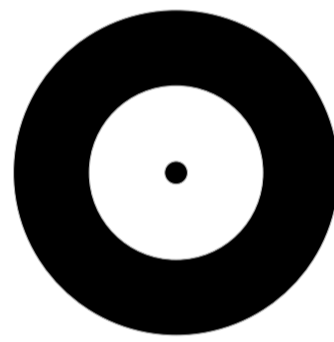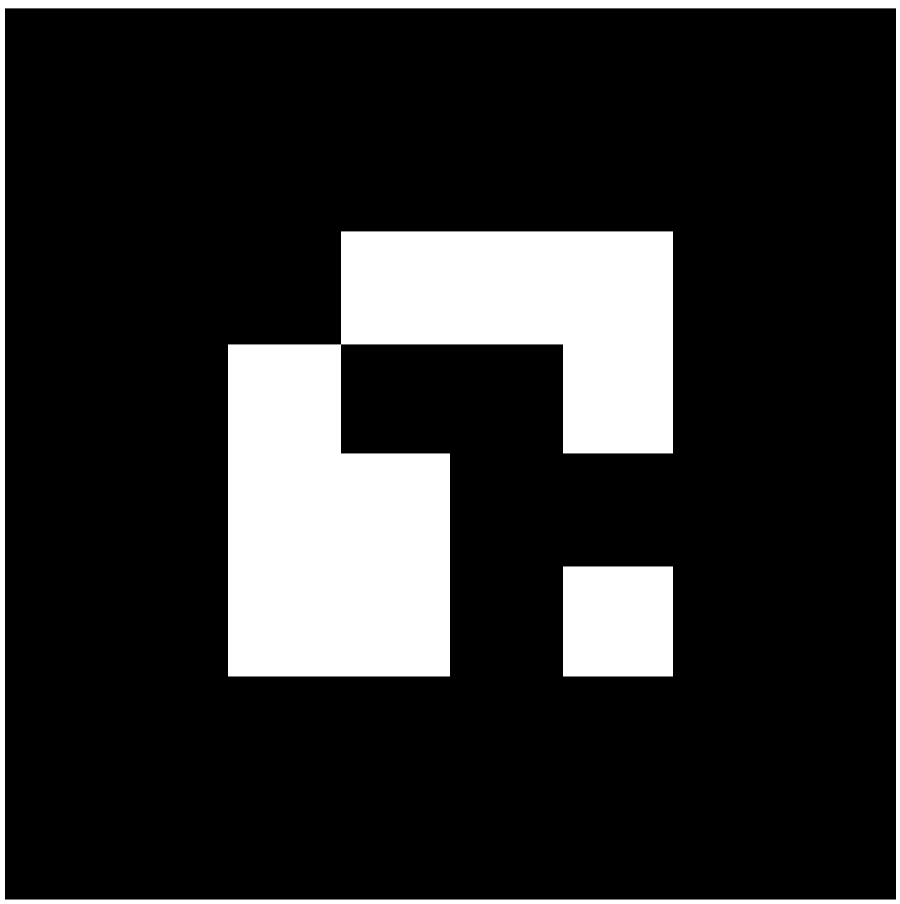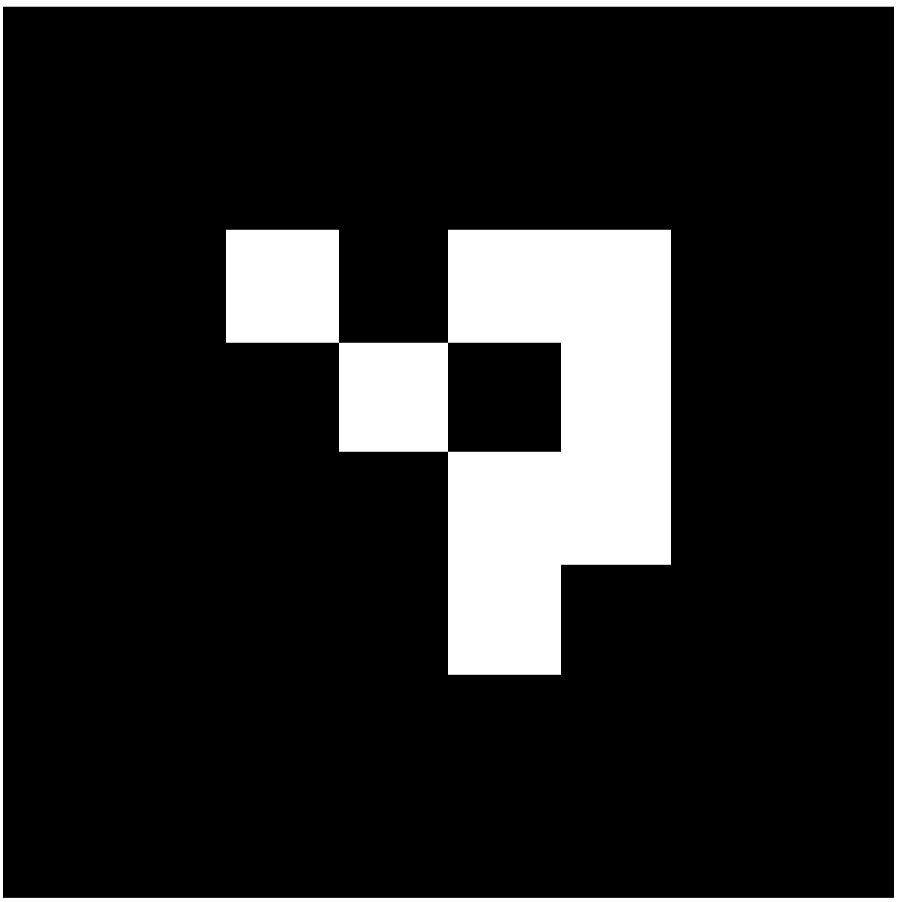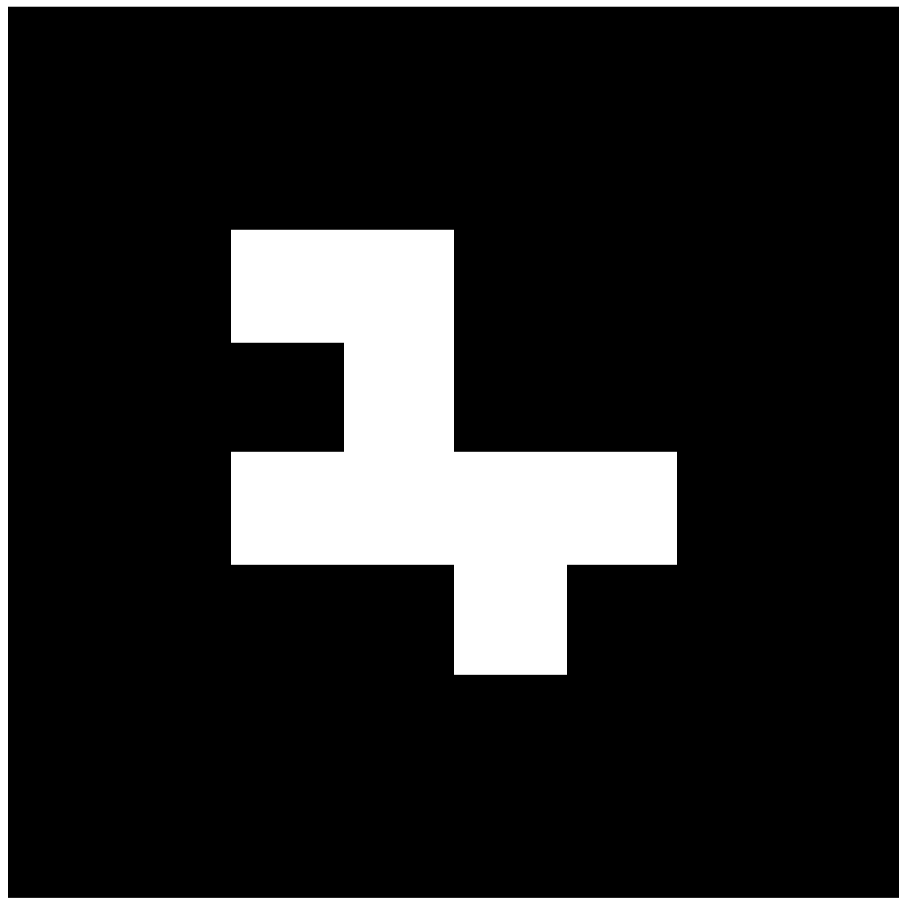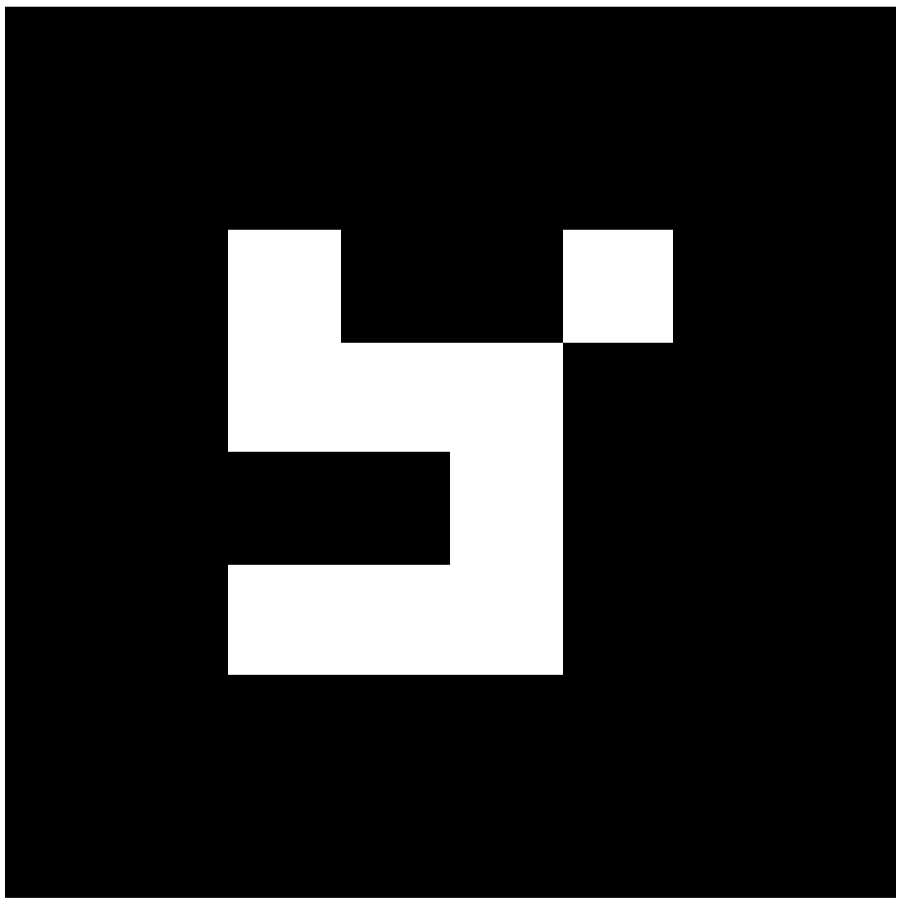

Supplement: Supplementary file 1 — (ZIP 35.2 KB) [file 13428_2019_1307_MOESM1_ESM.zip › stimulus_poster/poster_TobiiG2.pdf]
